# Assessing the efficacy of protected and multiple-use lands for bird conservation in the U.S.

**DOI:** 10.1371/journal.pone.0239184

**Published:** 2020-09-30

**Authors:** L. Lynnette Dornak, Jocelyn L. Aycrigg, John Sauer, Courtney J. Conway

**Affiliations:** 1 Department of Geography, University of Wisconsin-Platteville, Platteville, Wisconsin, United States of America; 2 Department of Fish and Wildlife Sciences, College of Natural Resources, University of Idaho, Moscow, Idaho, United States of America; 3 U.S. Geological Survey Patuxent Wildlife Research Center, Laurel, Maryland, United States of America; 4 U.S. Geological Survey, Idaho Cooperative Fish and Wildlife Research Unit, Department of Fish and Wildlife Sciences, College of Natural Resources, University of Idaho, Moscow, Idaho, United States of America; Sichuan University, CHINA

## Abstract

Setting land aside has long been a primary approach for protecting biodiversity; however, the efficacy of this approach has been questioned. We examined whether protecting lands positively influences bird species in the U.S., and thus overall biodiversity. We used the North American Breeding Bird Survey and Protected Areas Database of the U.S. to assess effects of protected and multiple-use lands on the prevalence and long-term population trends of imperiled and non-imperiled bird species. We evaluated whether both presence and proportional area of protected and multiple-use lands surrounding survey routes affected prevalence and population trends for imperiled and non-imperiled species. Regarding presence of these lands surrounding these survey routes, our results suggest that imperiled and non-imperiled species are using the combination of protected and multiple-use lands more than undesignated lands. We found no difference between protected and multiple-use lands. Mean population trends were negative for imperiled species in all land categories and did not differ between the land categories. Regarding proportion of protected lands surrounding the survey routes, we found that neither the prevalence nor population trends of imperiled or non-imperiled species was positively associated with any land category. We conclude that, although many species (in both groups) tend to be using these protected and multiple-use lands more frequently than undesignated lands, this protection does not appear to improve population trends. Our results may be influenced by external pressures (e.g., habitat fragmentation), the size of protected lands, the high mobility of birds that allows them to use a combination of all land categories, and management strategies that result in similar habitat between protected and multiple-use lands, or our approach to detect limited relationships. Overall, our results suggest that the combination of protected and multiple-use lands is insufficient, alone, to prevent declines in avian biodiversity at a national scale.

## Introduction

Reduction in habitat quantity and quality is the major cause of species extinctions and endangerment [1–4]. Hence, conservation efforts that effectively improve or maintain habitat and thereby prevent further losses to biodiversity are imperative. Perhaps the most common approach has been to conserve lands, where some or all types of anthropogenic activities or land uses are restricted (multiple-use vs. protected lands, respectively) [5–8]. This approach has been used for over two millennia [8, 9], often with the implicit assumption that these conserved lands protect biodiversity. However, explicit evidence that protected and multiple-use lands help to maintain species populations is limited at best [10]. To use resources efficiently, we should evaluate the extent to which all or part of these lands in the U.S. is working to preserve biodiversity and use this information to inform our management strategies [11–13]. Here, we evaluate the differences in use of, and associations between, protected and multiple-use lands within the contiguous U.S. and prevalence and population trends of birds across large spatial extents.

Lands managed to conserve biodiversity in the U.S. have been divided into two broad categories on the basis of the level of protection: (1) protected land—land designated with the explicit intent of protection of native land cover types often with the stated goal of preserving biodiversity, ecosystem services, and cultural values [14], and (2) multiple-use land—land protected from complete land cover conversion but managed for one or more extractive uses (e.g., grazing, logging, energy development) and where conservation of biodiversity is only one of many goals [15, 16]. In the contiguous U.S., the combination of these lands covers approximately 26% of the total land area and is imbedded in a matrix of lands with varying land-use intensities [17].

Although extensive, protected and multiple-use lands in the U.S. consists of thousands of discrete land parcels amalgamated through piecemeal designations stemming from political, social, and conservation objectives [6, 11, 18]. Moreover, these lands are managed by many different entities (i.e., local, state, and federal agencies as well as corporate, private, and non-profit organization land-holders), each having distinct conservation and land-use objectives [11, 19]. Time and resources are devoted to the designation and management of the both protected and multiple-use lands with the implicit assumption that it is effectively conserving biodiversity. But, is this assumption valid? Is this management achieving its intended objective?

Past efforts to evaluate the efficacy of the protected and multiple-use lands in conserving biodiversity examined the extent to which these lands represent species (e.g., species richness and abundance; [20, 21]) or ecological systems (e.g., the percent of each vegetative community that is protected; [11, 12]). However, equal representation of species within these lands does not tell us whether all species benefit. In other words, species may be equally represented, but their populations may be faring no better than populations outside protected and multiple-use lands [22, 23]. Several recent studies [24–27] have reported that protected and multiple-use lands provide protection of natural land cover, which in turn appears to protect bird species (by improving species abundance and/or occurrence). However, with the exception of Wood et al. [20, 21], these studies were restricted spatially, focused on single species or small species groups, and/or occurred outside the U.S.

A more direct and informative approach to evaluate the efficacy of protected and multiple-use lands is to compare species population trends and prevalence among protected, multiple-use, and undesignated lands (i.e., land with no designated level of protection or management; [21, 28]) at the continental scale. Birds are considered good indicators of environmental health because they respond quickly to perturbations [29, 30], and thus are useful taxa for this evaluation. The North American Breeding Bird Survey (BBS; [31]) is a taxonomically broad (i.e., population trends can be estimated for 548 species), long-term (i.e., >50 years), and geographically extensive (continent-wide) survey data of birds in North America. This big data survey, through its strict standards and consistent methodologies, provides an appropriate avian dataset for evaluating bird population changes on protected areas [32].

Populations of many bird species in the U.S. have declined and those declines are often attributed to the loss, degradation, and fragmentation of habitat [33–36]. We were specifically interested in comparing the effectiveness of protected and multiple-use lands for two groups of bird species in the U.S.: imperiled species and non-imperiled species. Our objectives were to determine for these bird groups whether: (1) there was an effect of *presence* of protected and multiple-use lands (i.e., the presence of protected, multiple-use, or undesignated lands influenced species prevalence and population trend); and (2) there was an effect of *proportion of area* of protected and multiple-use lands (i.e., the proportional area of protected and multiple-use lands influenced species prevalence and population trend). We hypothesized that imperiled species prevalence should be higher on, and population trends should be positively correlated with, protected lands more than multiple-use or undesignated lands. These hypotheses are grounded on the explicit but untested assumption that protected land is preserving avian biodiversity better than multiple-use or undesignated lands.

## Materials and methods

### Data description

We used data from the USGS BBS and the USGS Gap Analysis Project (USGS-GAP) Protected Areas Database of the U.S. (PAD-US) to compare long-term population trends and species prevalence among protected, multiple-use, and undesignated lands in the U.S. All spatial data layers were projected to USA Contiguous Albers Equal Area Conic USGS (North American 1983 datum). We used the PAD-US (version 1.3; [17]) to differentiate between protected land, multiple-use land, and undesignated land. PAD-US is an inventory of public and private lands (i.e., fee-owned land and conservation easements) classified into four protection categories based on protection status and land management intent [37]. Status 1 applies to lands with permanent protection from natural land cover conversion and managed specifically for biodiversity. Status 2 lands are like Status 1 lands except that they may have management practices that degrade the quality of existing natural communities, including suppression of natural disturbances. Status 3 lands are those where activities that cause permanent land cover conversion are prohibited but management intent includes multiple-use objectives (e.g., timber harvest, energy development, recreation, and conservation). Status 4 lands have no known protection from land cover conversion and management intent is either not specified or unknown [37]. Status 1 and 2 lands confer the highest level of protection, followed by Status 3 and Status 4 lands. For our analyses, we refer to Status 1 and 2 lands as ‘protected’ land [11, 18], Status 3 lands as ‘multiple-use’ land, and Status 4 lands as ‘undesignated’ land.

#### Breeding Bird Survey data

To evaluate the efficacy of protected and multiple-use lands, we used count data from the BBS across the contiguous U.S. (CONUS). The BBS is a long-term (> 50 years), continent-wide monitoring program that provides standardized survey data for breeding bird populations on >4,000 roadside survey routes. Most BBS routes are surveyed annually, and observers count the number of birds detected for each species at 50 sampling points at 0.8-km intervals along a 39-km survey route [31].

**Fig 1:**
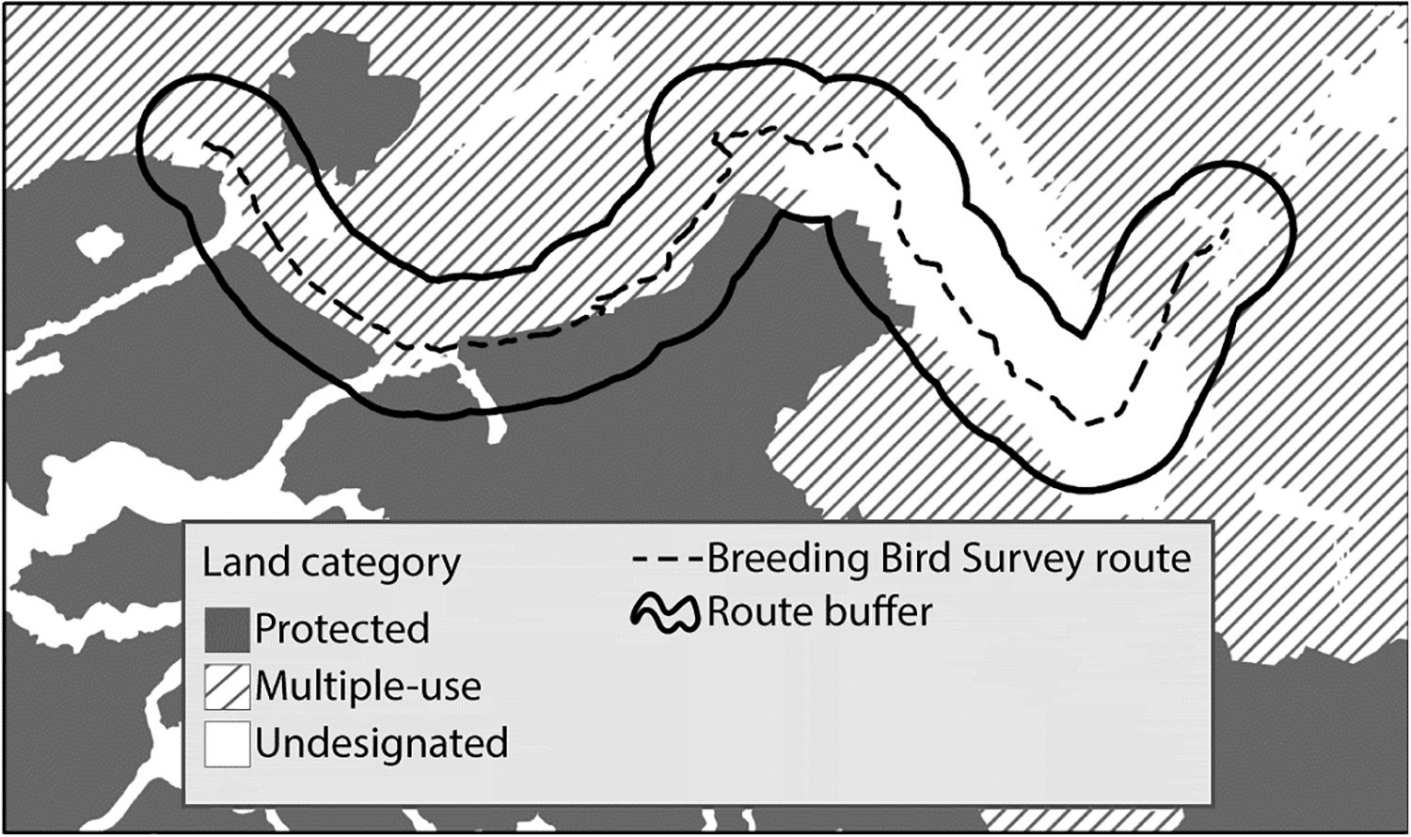
Example of Breeding Bird Survey (BBS) route with 2000-meter radius buffer. Protected (gray), multiple-use (hatched), and undesignated lands (white) are shown with the BBS route (dashed line) and the route buffer (solid line).

We delineated a 2000-m radius buffer (hereafter, ‘buffer’) around each BBS survey route that met our criteria for assessing prevalence and population trend (see section below, Fig 1) and used ArcGIS 10 (Environmental Systems Research Institute, Redlands, California, USA) to calculate the following attributes within each buffer: percent of protected land, percent of multiple-use land, percent of undesignated land, median elevation, and latitude and longitude at the buffer centroid. Our goal was to assess whether the landscape matrix surrounding each BBS route (i.e., the landscape to which the local bird population was exposed) affected species prevalence and population trend. Although most birds detected during BBS surveys are <400 m from the surveyor [31], we used a 2000-m radius buffer on each side of the route (rather than a 400-m buffer) because we assumed that birds detected within 400 m had access to and were influenced by areas much beyond 400 m. A 2000-m radius is large enough to capture the characteristics of the landscape matrix and significant associations that exist between bird populations on routes and the landscape without creating overlap between adjacent route buffers [38–42].

Protected and multiple-use lands are not evenly nor randomly distributed across the U.S. Hence, the proportion of each of the three protection categories varied widely among BBS route buffers. Also, some bird species occurred on only a few BBS routes. As such, we limited our analyses to bird species for which we could make adequate inferences by including only bird species that met minimum criteria for each objective. Details are given below in the *Statistical Analysis* section.

The proportions of protected and multiple-use lands within the BBS route buffers we used (7% for protected land and 18% for multiple-use land across all buffers) were nearly identical to their proportions across CONUS (7% for protected land and 19% for multiple use land) based on PAD-US [17]. This indicates that the proportion of protected and multiple-use lands within the BBS route buffers are representative of the proportion of these areas across CONUS.

#### Bird species

We defined imperiled species as those whose populations are declining enough to warrant recognition by national or international organizations. Hence, we identified imperiled species as those that appeared on one or more of the following three lists: U.S. Fish and Wildlife Service Birds of Conservation Concern [43]; International Union for Conservation of Nature Red List bird species that met the criteria for ‘vulnerable,’ ‘critically threatened,’ or ‘endangered’ [44]; and Partners-in-Flight Common Species in Steep Decline [33]. We defined non-imperiled species as those that were not included on any of the three lists above. We overlaid BBS routes selected for inclusion in our analysis with their buffers onto a map of ecological communities from the National GAP Land Cover database, version 2 [45]. Imperiled species were detected within BBS route buffers within all ecological communities (e.g., National Vegetation Classification classes; forest and woodland, semi-desert, etc.) and represented diverse taxonomic groups (228 species, 26 families, 12 orders). We note that most boreal species are excluded from these groups because the extent of our analysis is limited to the CONUS.

#### Spatial and temporal subsets

Because protected and multiple-use lands are not evenly distributed across CONUS, we created western and eastern subsets (i.e., West and East; dividing the BBS routes at the 98^th^ meridian) to assess whether our results differed regionally. This split allowed us to examine whether there were regional differences in the efficacy of protected and multiple-use lands while still meeting our criteria for minimum species frequency (see below). Splitting the data further into smaller ecological or conservation regions, as in Wood et al. [20, 21], yielded too few species to produce a robust analysis. Furthermore, because BBS routes were surveyed inconsistently at various times during 1966–2014, we created a short-term dataset (1993–2014) to examine whether our results differed among these two temporal subsets (i.e., 1966–2014 and 1993–2014). The short-term dataset is consistent with a temporal grouping used by the BBS [46]. We analyzed these subsets to examine, qualitatively, any temporal or spatial bias in our data.

#### Prevalence and population trends

**Fig 2:**
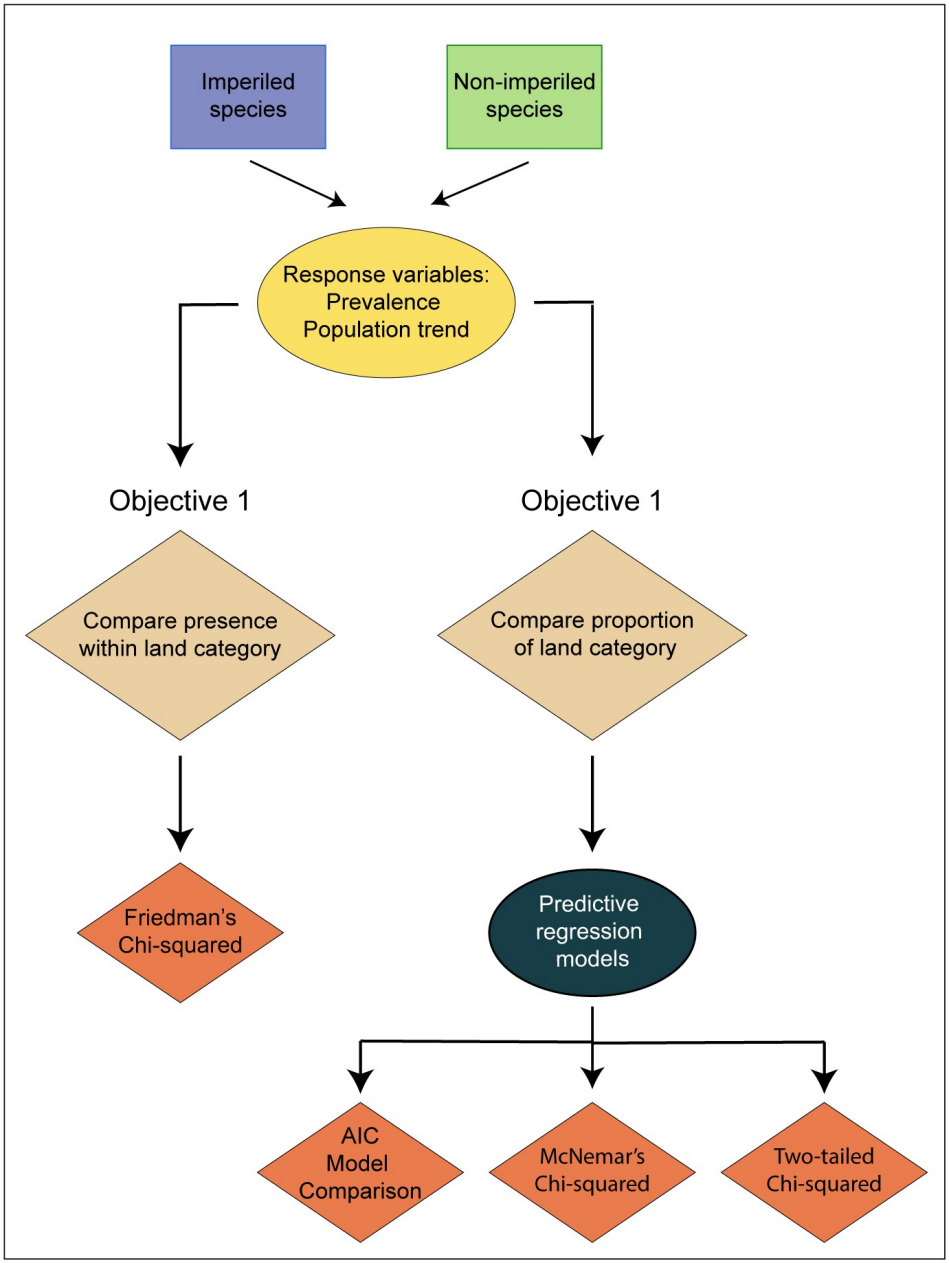
Illustration of the study objectives and statistical analyses. Objective 1 evaluated if the presence of protected, multiple-use, or undesignated land in the 2000-meter radius buffer surrounding Breeding Bird Survey (BBS) routes influenced prevalence and population trends of imperiled or non-imperiled bird species. The effect was tested with the Friedman’s chi-square test. Objective 2 evaluated if the proportional area of protected and/or multiple-use lands in the buffer surrounding BBS routes influenced prevalence and population trends of imperiled or non-imperiled bird species. Multivariate regressions with three statistical methods (AIC model comparison, McNemar’s chi-squared test, and a two-tailed chi-squared test) were used for this objective.

To assess the efficacy of protected and multiple-use lands, we examined two metrics related to species persistence: prevalence and population trend (Fig 2). We defined prevalence as the consistency with which presence of an individual species was detected on a route across years. We used 50-count stop data downloaded directly from the BBS website (https://www.pwrc.usgs.gov/bbs) and calculated prevalence as the number of years an individual species was detected on a route divided by the number of years the route was surveyed (i.e., the proportion of years a species was detected on a BBS route). Effectively, prevalence, as we have estimated it here, is occupancy without adjustment for detection. We calculated prevalence only for species that were detected on ≥10 BBS routes. We chose a minimum of 10 routes as a conservative estimate of detection of a species on a route. For example, in 10 routes, there is 97% chance of observing a species, even with a 5% chance of detecting it at any stop.

We estimated route-specific population trends for each species [47, 48]. However, we only estimated trends for routes that met two criteria: (1) counts for a species had to occur in at least two 10-year periods during 1966–2014, to ensure that the trend estimates spanned the time interval; and (2) the routes had to be surveyed ≥10 years during 1966–2014. We used a Poisson regression with log links for the trend estimates for each route and included covariates to control for observer effects. The slope parameter associated with year, exponentiated, was the trend estimate [49, 50].

During 1966–2014, the amount (i.e., total area) of protected land has increased in the U.S. [17]. However, much of the land initially given protected status between 1966–2014 was already protected from permanent land conversion (e.g., BLM lands identified as Wilderness Study Areas after implementation of the Federal Lands Policy and Management Act in 1976; [16, 18]) and likely contained conditions and species worthy of inclusion in protected lands prior to formal designation. Therefore, we assumed that changes in protected land designations minimally influenced bird prevalence and population trend on BBS routes during 1966–2014.

### Statistical analyses

For all spatial analyses, we used ArcGIS 10.x (Environmental Systems Research Institute, Redlands, California, USA) and for all statistical analyses we used R (R Development Core Team, version 3.4.0). An alpha of 0.05 was used for evaluation of statistical significance for all tests unless otherwise noted. Individual tests are described below in their respective sections.

#### Objective 1: Effect of presence of protected, multiple-use, and undesignated lands

To address our first objective, we tested whether species prevalence or population trend differed among routes embedded primarily in protected, multiple-use, and undesignated land for imperiled and non-imperiled species (Fig 2). That is, we examined the effect of the land category (protected, multiple-use, or undesignated) that dominated the landscape within each route buffer on both prevalence and population trends. We categorized a route buffer as being “primarily embedded” if ≥50% of the route buffer was composed of any single protection category (Fig 1).

We estimated mean prevalence and mean population trend for each species across years within each of three categories of BBS routes: (1) ≥50% of the buffer within protected land, (2) ≥50% of the buffer within multiple-use land, or (3) ≥50% of the buffer within undesignated land. We only included species that had routes representing each of these three categories. We calculated the medians and standard errors of the prevalence and population means, respectively. We used Friedman’s test with post-hoc analyses [51] to evaluate the difference among the three categories of BBS routes (protected, multiple-use, and undesignated) for both imperiled and non-imperiled species. Friedman’s test is a non-parametric alternative to the one-way ANOVA with repeated measures. All assumptions for this test were met. We used α = 0.1 for all comparisons except for the post-hoc analyses, where we used a Bonferroni adjusted α = 0.03 (i.e., ~0.1/3 post-hoc comparisons [52]).

#### Objective 2: Effect of the proportional area of protected and multiple-use areas

We used three methodological approaches to examine whether the proportional area of protected or multiple-use land affected either species prevalence or population trend among the BBS route buffers (Fig 2, also see below). To estimate proportional area, we calculated the area of protected and multiple-use lands within each route buffer. For each approach, we used multiple linear regressions to evaluate the effects of land category (i.e., protected or multiple-use) on species prevalence and population trend for both imperiled and non-imperiled species groups.

We regressed both prevalence and population trend against the proportion of the route buffer area (i.e., proportional area) that was protected and multiple-use lands. We also included median elevation, total years the route was surveyed, longitude and latitude at the buffer centroid, proportion of developed land [45], and proportion of agricultural land [45] as covariates in the regression models to assess the effects of the land category (i.e., protected or multiple-use) on the response variables (Eq 1). We included the total years the route was surveyed as a potential explanatory variable because we assumed that the number of years available to estimate prevalence and population trend would affect the variation in these metrics and, hence, might affect our ability to detect a relationship with land category. We used these regression models to assess the strength and direction of the relationship between proportional area of protected and multiple-use lands and both prevalence and population trend. We included only the proportional area of protected and multiple-use lands within the buffers in this analysis (and not the proportional area of undesignated land) because including the proportional area of all three categories (protected, multiple-use, and undesignated lands) creates model instability that can arise with lack of independence among explanatory variables and the proportion of all three categories sums to 1.0 [51] None of our explanatory variables were highly correlated (r <0.60).

#### Eq 1. Regression model for predictors of trend

The analysis of prevalence used the same model.

$$Trend{=}_{p}Protected{+}_{p}Multiple-use{+}_{p}Developed{+}_{p}Agriculture{+}_{n}Years\;surveyed+Median\;elevation+Longitude+Latitude$$ (1)

We limited our regression analysis to include only bird species for which we had sufficient data. To do this, we only included species in the regression analysis for which ≥25% of the BBS routes where the species was detected had buffers consisting of ≥5% of either protected or multiple-use lands. Thus, we required that a species occur with a minimum frequency on routes whose buffers contained a minimum proportional area of protected or multiple-use land.

#### AIC model comparison

We assessed the overall influence of the predictor variables of interest (i.e., proportional area of protected and multiple-use lands) on the response variables (i.e., prevalence and population trend) by comparing multiple linear regression models using Akaike Information Criterion (AIC; Fig 2). Unlike McNemar’s chi-squared test (described below), we included species with any association (positive or negative) with proportional area of protected and multiple-use lands. We examined whether multiple linear regression models better explained the variation among BBS routes with regards to prevalence and population trend of bird species when the proportional area of protected and multiple-use lands within the buffer surrounding the BBS routes was included. We used AIC to compare models with and without proportional area of protected and multiple-use lands within the buffer. We defined top-competing models as those with ΔAIC values <2 [53].

#### McNemar’s chi-squared test

We used McNemar’s chi-square test to determine if the percent of bird species with prevalence and population trend positively associated with the proportional area of protected land within the buffer was greater than the percent of species with prevalence and population trend positively associated with the proportional area of multiple-use land (Fig 2). We examined these effects for imperiled and non-imperiled species independently. We used McNemar’s chi-squared test for non-independent samples because prevalence and population trends for some species were positively associated with both proportional area of protected and multiple-use lands [51].

#### Two-tailed chi-squared test

We used a two-tailed chi-square test to determine which land category was more positively associated with prevalence and population trend. Here, we examined the regression coefficients, associated with proportional area of protected and multiple-use lands within route buffers, to determine which land category was more positively associated with prevalence and population trend (Fig 2). We did this for both imperiled and non-imperiled species. The more positive the coefficient, the greater the effect of protected and multiple-use lands on prevalence and population trend (i.e., a species may have a negative coefficient associated with proportional area of protected land, but the magnitude of that coefficient may be less negative). Unlike McNemar’s chi-squared test (described above), we included species with any association (positive or negative) with proportional area of protected and multiple-use lands. For example, a species may have a negative coefficient value associated with proportional area of protected land but that negative coefficient for proportion of protected land may be less negative than the coefficient for the proportion of multiple-use land (i.e., the species may be faring better on protected land despite a pervasive decline in prevalence or population trend). Here, we used a two-tailed test of proportions with a null hypothesis that the proportion was 0.5 [51].

## Results

### Spatial and temporal subsets

As a result of the criteria applied to each dataset, and as an artifact of the differences between the datasets, the total species per analysis group was reduced (Table 1). Sub-setting the data spatially and/or temporally further reduced the number of species and routes included in the analysis (Table 1). The smaller sample sizes, subsequently, restricted our ability to draw broad comparisons between subsets. Therefore, we report the entire dataset (CONUS and 1966–2014) for all our analyses of prevalence and population trend to limit inherent problems associated with reducing route and species sample sizes (i.e., too few routes and/or too few species). For analyses of the spatial and temporal subsets, see the supporting information (cited with each objective and its associated analysis).

**Table 1:** Number of species and Breeding Bird Survey (BBS) routes used for each analysis. Data are presented by objective, metric, and species group as well as by spatial and temporal subset.

Analysis	Metric	Species Group	Long-term^a^			Short-term^b^		
			CONUS^c^	West^d^	East^d^	CONUS	West	East
Objective 1	Prevalence	Imperiled species	43	17	27	33	12	20
		Non-imperiled species	124	57	78	100	37	68
		BBS routes	2850	1020	1830	2333	846	1487
	Population trend	Imperiled species	61	28	35	56	19	31
		Non-imperiled species	161	93	107	157	81	105
		BBS routes	2913	1052	1861	2404	873	1531
Objective 2	Prevalence							
		Imperiled species	61	52	25	57	49	24
		Non-imperiled species	134	116	52	127	114	55
		BBS routes	2398	1002	1419	1977	829	1187
	Population trend	Imperiled species	75	59	35	73	55	31
		Non-imperiled species	156	143	67	167	141	68
		BBS routes	2706	1059	1638	2243	884	1327

^a^ Long-term data are BBS survey data from years 1966–2014.

^b^ Short-term data are BBS survey data from years 1993–2014.

^c^ CONUS data indicates data from across the contiguous US.

^d^ West and East routes were divided at the 98^th^ meridian.

### Objective 1: Effect of presence of protected, multiple-use, and undesignated lands

**Fig 3:**
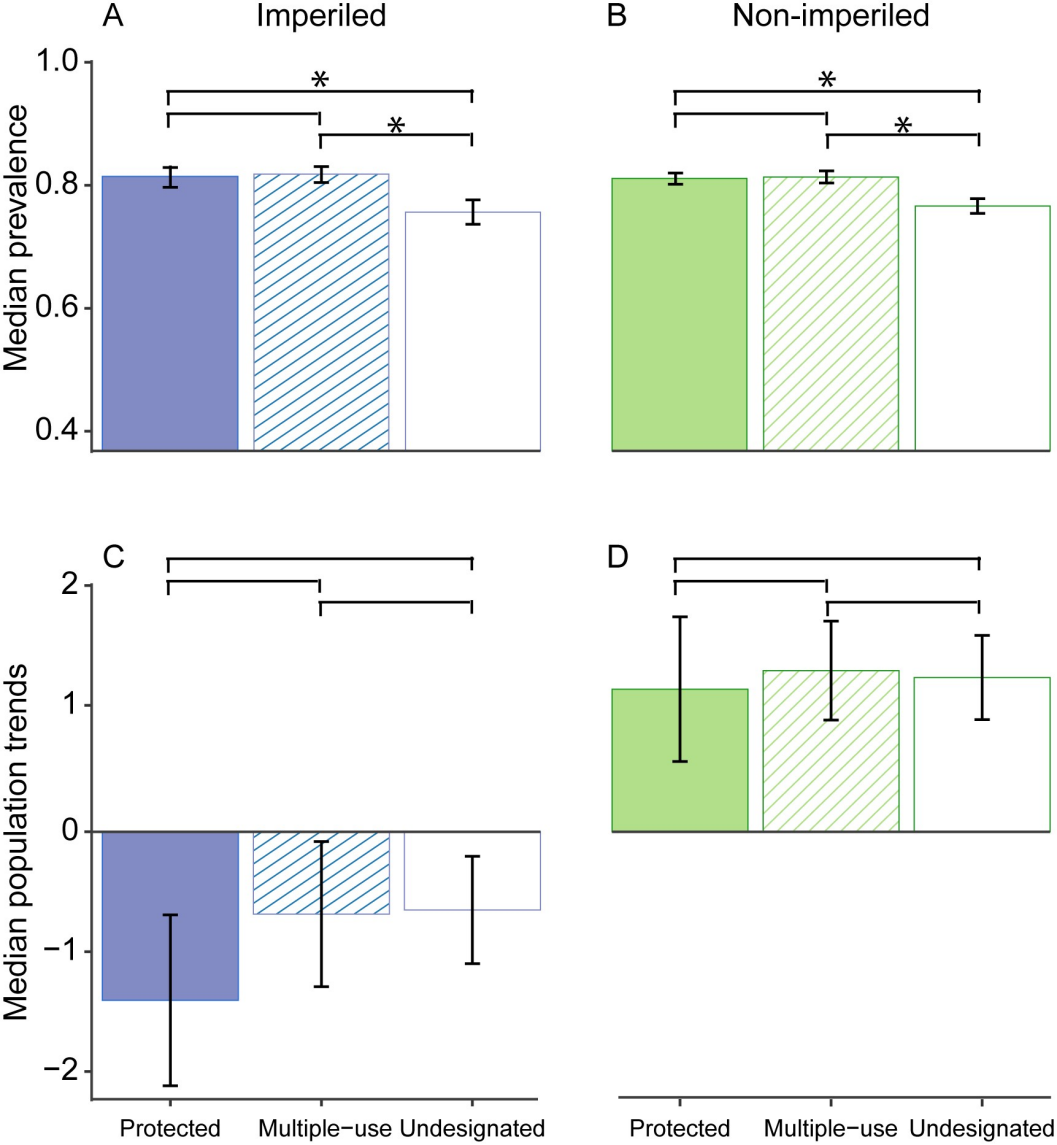
Median prevalence and population trends based on the presence of protected, multiple-use, and undesignated lands. Median prevalence (A and B) and median population trends (C and D) for imperiled species (*n* = 61 for median prevalence, *n* = 75 for median population trends) and non-imperiled species (*n* = 134 for median prevalence, *n* = 156 for median population trends) for Breeding Bird Survey (BBS) routes with ≥50% of protected, multiple-use, or undesignated land within a 2000-meter radius buffer surrounding routes. Brackets over bars show pairewise comparisons (e.g., the top bar shows the protected vs. undesignated comparison). Asterisks indicate significant differences between pairs based on Friedman’s chi-square test with post-hoc analysis. Significance was evaluated with p ≤ 0.10 for Friedman’s chi-square tests and p ≤ 0.03 with Bonferroni adjustment for post-hoc analysis. Error bars respresent ± SE.

Median prevalence differed among BBS routes based on the presence of protected, multiple-use, and undesignated lands within the route buffer for both imperiled (*n* = 61, χ^2^(2) = 11.4, DF = 2, p = 0.003) and non-imperiled (*n* = 134, χ^2^(2) = 28.0, DF = 2, p < 0.001) species. Both species groups were more prevalent on BBS routes embedded in protected (imperiled: p *=* 0.014 and non-imperiled: p < 0.001, respectively) and multiple-use (imperiled: p = 0.007 and non-imperiled: p < 0.001, respectively) lands than on routes embedded in undesignated land (Fig 3A and 3B). We found no difference in prevalence between BBS routes embedded in protected and multiple-use lands for either imperiled species (p *=* 0.975; Fig 3A) or non-imperiled species (p = 0.923; Fig 3B). See S1 Fig and S4 Table for analysis of data subsets.

Median population trends of imperiled species were negative (and lower than those of non-imperiled species), regardless of whether the BBS route was embedded primarily in protected, multiple-use, or undesignated lands (Fig 3C). We found no difference in median population trends among the three categories (i.e., protected land, multiple-use lands, undesignated land) on BBS routes for either imperiled (*n* = 75, χ^2^ = 1.6, DF = 2, p = 0.455) or non-imperiled species (*n* = 156, χ^2^ = 0.6, DF = 2, p = 0.742; Fig 3C and 3D). See S2 Fig and S4 Table for analysis of data subsets.

### Objective 2: Effect of the proportional area of protected and multiple-use areas

Regression models assessed the strength and direction of the relationship between proportional area of protected and multiple-use lands and both prevalence and population trend. The relationships between the proportional area of protected and multiple-use lands and both prevalence and population trend varied among bird species (S2 and S3 Tables).

#### AIC model comparison

**Fig 4:**
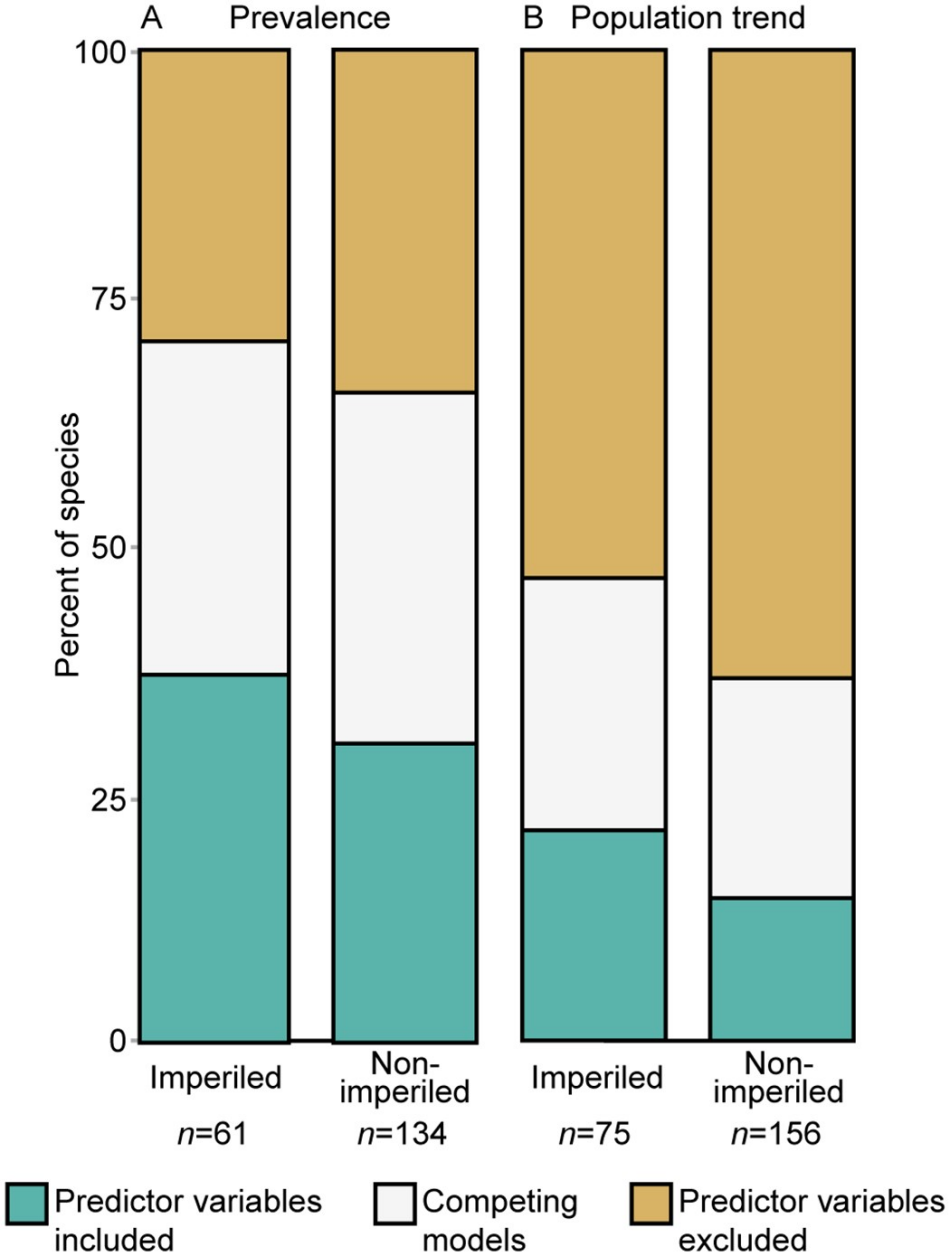
Comparison of linear models that included or excluded predictor variables. Data for prevalence (A) and population trends (B) are presented. ‘Predictor variables included’ indicates models which included proportional area of protected and multiple-use lands as predictor variables in the linear regression models to explain prevalence and population trends. ‘Competing models’ were those with delta AIC values <2, and models in which including or excluding the predictor variables did not change the delta AIC value. ‘Predictor variables excluded’ indicates models that did not include the proportional area variables. Additional covariates included proportion of developed areas, proportion of agricultural areas, median elevation of BBS route, total number of years BBS route was surveyed, and longitude and latitude at centroid of buffer.

The proportional area of protected and multiple-use land within route buffers helped to explain variation in prevalence for 38% of imperiled species and 31% of non-imperiled species (Fig 4A). Similarly, the proportional area of protected and multiple-use lands within route buffers helped explain more of the variation in population trend for imperiled species (21%) than non-imperiled species (14%). That is, the predictor variables of main interest (i.e., proportional area of protected and multiple-use lands) are included in the top model for more imperiled species than they are in the top model for non-imperiled species; this is true for both prevalence and population trends (Fig 4A and 4B). See S3 and S4 Figs for analysis of data subsets.

#### McNemar’s chi-squared test

**Fig 5:**
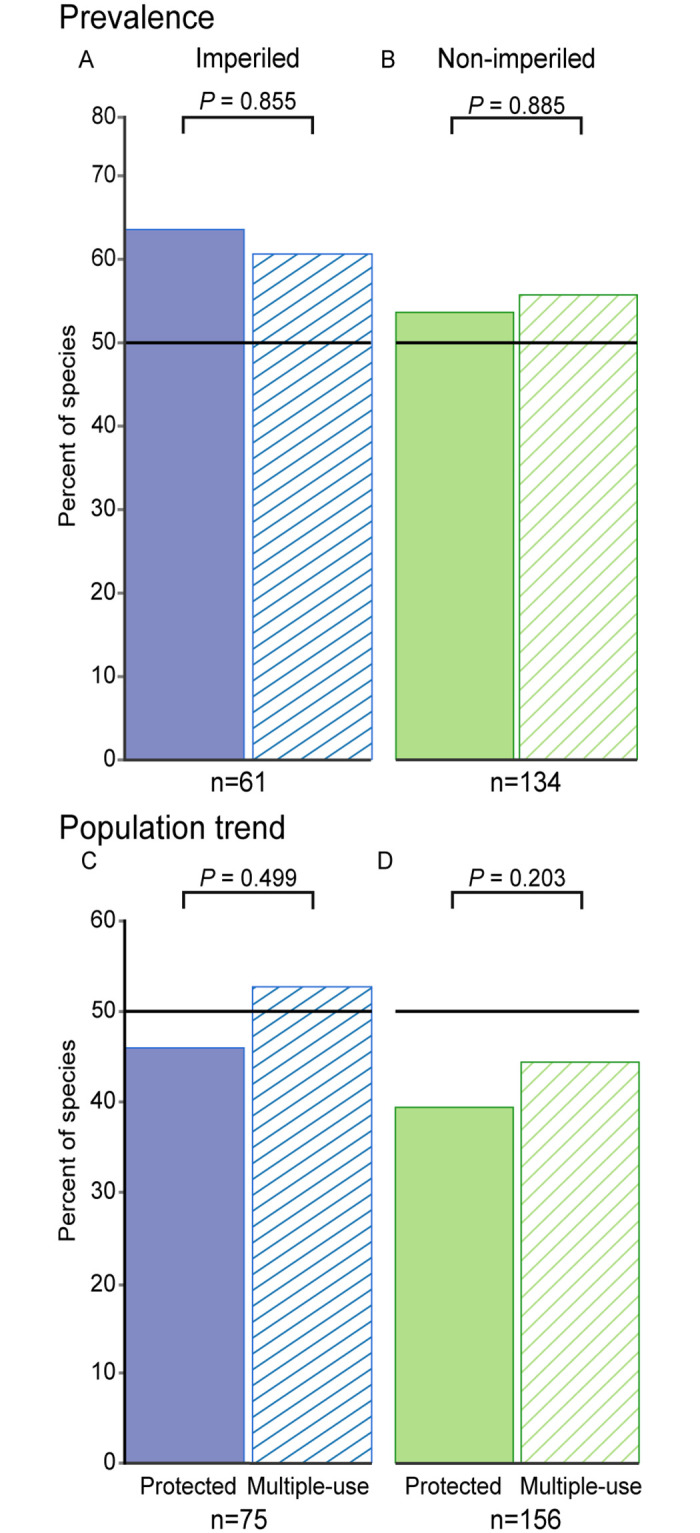
Percent of species for which prevalence was positively associated with land category. Prevalence (A and B) and population trend (C and D) associations are presented for both imperiled and non-imperiled species. Proportional area was calculated within a 2000-meter radius buffer surrounding Breeding Bird Survey (BBS) routes. Horizontal lines at 50% indicate the null model (i.e., the percentage we would expect if bird populations were not influenced by proportional area of protected or multiple-use land within the buffer surrounding the BBS route). Significance was evaluated with *P* ≤ 0.10 for McNemar’s chi-squared tests.

We detected no difference between the percent of species for which prevalence was positively associated with proportional area of protected land and the percent of species for which prevalence was positively associated with proportional area of multiple-use land for either imperiled (McNemar’s χ^2^ = 0.03, p = 0.855) or non-imperiled species (McNemar’s χ^2^ = 0.02, p = 0.885; Fig 5A and 5B). The percent of bird species for which prevalence was positively associated with the proportional area of protected land or multiple-use land in the buffer was 64% and 61%, respectively, for imperiled species and 54% and 55%, respectively, for non-imperiled species (Fig 5A and 5B). See S5 Fig and S5 Table for analysis of data subsets.

The percent of bird species for which population trend was positively associated with the proportional area of protected land or multiple-use land was 47% and 53%, respectively, for imperiled species and 39% and 46%, respectively, for non-imperiled species (Fig 5C and 5D). However, we found no difference in the percent of species for which population trend was positively associated with protected and multiple-use lands for imperiled (McNemar’s χ^2^ = 0.46, p = 0.499) or non-imperiled species (McNemar's χ^2^ = 1.62, p = 0.203; Fig 5C and 5D). See S6 Fig and S5 Table for analysis of data subsets.

#### Two-tailed chi-squared test

For the analysis of prevalence, only 53% of imperiled species and 49% of non-imperiled species had regression coefficients for protected land that were more positive than regression coefficients for multiple-use land. We found no difference in percent of species in either imperiled (χ^2^ = 0.07, p = 0.798) or non-imperiled (χ^2^ = 0.07, p = 0.796) species, indicating that the effect of protected land is the same as that of multiple-use land regarding species prevalence. See S7 Fig and S6 Table for analysis of data subsets.

For the population trend analysis, we found no difference in imperiled species (χ^2^ = 0, p = 1). However, we did detect a statistical difference in non-imperiled species (χ^2^ = 2.83, p = 0.093); that is, more non-imperiled species were positively associated with multiple-use land compared to those associated with protected land. Fifty-one percent of imperiled species and 43% of non-imperiled species had protected land regression coefficients that were more positive than the regression coefficients for multiple-use land. See S8 Fig and S6 Table for analysis of data subsets.

## Discussion

Our results suggest that both imperiled and non-imperiled species are using protected and multiple-use land with higher prevalence than undesignated lands. This result supports the implicit (but seldom tested) assumption that both of these land categories help prevent extinction and maintains avian biodiversity. However, our hypothesis that species prevalence should be higher and population trend should be positively correlated with protected land more than multiple-use or undesignated lands was not well supported. We found that imperiled species have negative long-term population trends, even on routes primarily embedded in protected and multiple-use lands (Fig 3). Additionally, although some species responded favorably to the proportional area of protected and multiple-use lands along the BBS routes, we found little evidence that protected lands do better than multiple-use lands to sustain population trends or to maintain species use of these areas, either for imperiled or non-imperiled species (Fig 5).

### Objective 1: Effect of presence of protected, multiple-use, and undesignated lands

We detected a difference between protected and undesignated lands for species prevalence for both imperiled and non-imperiled species. This difference may be attributed to several factors: (1) sample size of species or BBS routes, (2) total area of protected land that intersected the routes, (3) types of land-use in undesignated lands, and/or (4) the permanence of land cover types on protected lands [8, 54]. In contrast to our results, trends in species prevalence have not been found to differ between protected and ‘unprotected’ lands in wet tropical forests in Australia [55]. However, our study had a larger spatial extent (9,857,000 km^2^ vs. 9,000 km^2^), a longer time period (49 years vs 14 years), a larger and more diverse group of species (222 vs. 21) and included a wider suite of vegetative communities compared to Barnes et al. [55]. For example, when we examined subsets of our long-term CONUS dataset, we also found no difference in prevalence between protected (or multiple-use) and undesignated lands (*n* = 20 for imperiled species; S1 Fig and S4 Table). These short-term, eastern and western datasets had reduced extents, fewer species, and fewer represented vegetative communities. Thus, our large sample size over a longer period and large spatial extent likely improved our ability to detect a difference in prevalence between protected and undesignated lands.

The persistent negative population trends for imperiled species regardless of the land category surrounding the BBS routes is concerning. Populations of imperiled species appear to be faring no better on protected lands than they are on undesignated lands. Thus, our results suggest that protected lands cannot guarantee protection from population declines or extinction [56, 57]. However, some studies have demonstrated reduced risk of extinction or slowing of population declines within protected lands [5, 56]. It is possible that the effect of protected lands may take time to be realized. Our short-term analyses, which included only more recent BBS data, showed slightly more positive population trends (compared to the long-term datasets) for imperiled species on routes primarily embedded in protected and multiple-use lands (S2 Fig). This result could be an artifact of fewer species used in the analysis. However, it could also suggest that the effect of protected lands takes time to conserve populations because it is only detected recent years.

### Objective 2: Effect of the proportional area of protected and multiple-use areas

Protected and multiple-use lands benefited many species (imperiled and non-imperiled alike), as evidenced from the percent of species with prevalence and population trends that were positively associated with the proportional area of both land categories (Fig 5). These results concur with that of Wood et al. [21], who suggested that protected areas maintain natural land cover enough to source regional metapopulations. However, the effects of land categories on species were indistinguishable in our study. Why are protected lands not maintaining higher prevalence and populations trend compared to multiple-use lands? There are several possible hypotheses.

First, external pressures in the landscape matrix surrounding protected lands may compromise their integrity and function, thus diminishing their conservation benefit [20, 21, 58, 59]. Although protected lands are shielded from land use conversion [11, 37], they still may suffer the negative effects of pressures emanating from outside their boundaries, on both local (e.g., human encroachment, habitat fragmentation, biotic homogenization, invasive species; [58–61]) and global scales (e.g., climate change, human pressure; [5, 54]). Wood et al. [20, 21] showed that housing development within and surrounding protected lands negatively affected abundance and the structure of the bird community. Other studies, presented similar effects of these pressures expressed as lower bird species richness or population declines [57, 62]. Such effects may be dampened by multiple-use lands providing a buffer (e.g., the Stanislaus and Sierra National forests on two sides of Yosemite National Park; [63]).

In our analysis, only eight (~0.3%) routes had buffers completely composed of protected land (S1 Table). These routes occurred solely within national parks (Everglades, Death Valley, Big Bend, Yellowstone, and Glacier National Parks). Thus, the landscape beyond the route buffer may cushion the effects of external pressures for these routes for some species. Yet, even on these routes, the mean trends for imperiled species varied between 9 and -15. However, >99% of BBS routes are embedded in a heterogeneous matrix. Our results may indicate external pressures, like those surrounding the parks (e.g., human encroachment from gateway cities; [20, 64]), are negatively affecting bird populations to the extent that conservation efforts on protected lands are no more effective than within multiple-use lands. Species that use multiple-use lands may also experience external pressures; however, these effects may be lessened by the size of these lands (see below).

Second, size of a protected lands may affect their capacity to offer suitable home range area as well as buffer the effect of external pressures [57, 61, 64]. The largest, contiguous protected lands within CONUS managed by a single unit (e.g., National Park Service) are national parks (e.g., Death Valley and Grand Canyon, 1.2 and ~0.5 million ha, respectively; [17]) and wilderness areas (e.g., Frank Church-River of No Return Wilderness and Boundary Waters Canoe Area Wilderness, 0.9 and 0.4 million ha, respectively; [17]). Conversely, the largest, contiguous multiple-use lands managed by a single unit are BLM public lands (e.g., Nevada and Utah, 11.2 and 2.7 million ha, respectively; [17]) or national forests (e.g., Beaverhead-Deerlodge National Forest and Salmon-Challis National Forest, 1.35 and 1.2 million ha, respectively; [17]). Considering the areas of contiguous and adjacent lands (i.e., dissolving borders between adjacent parcels to evaluate contiguous areas with the same designation), multiple-use lands are larger (mean: 802 ha, median:16 ha) than protected lands (mean: 583 ha, median: 3 ha). Some species, such as the bald eagle (*Haliaeetus leucocephalus*) require ~2,160 ha during the breeding season and >1 million ha during the non-breeding season, and red-cockaded woodpeckers (*Dryobates borealis*) live in family groups that require ~80 ha each [65–67]. Many protected lands (e.g., state parks and wildlife management areas) may be too small to provide adequate habitat to affect long-term prevalence or population trends [68, 69]. Furthermore, the smaller the area of the protected land, the stronger the effects of external pressures [61]. Smaller protected lands may not be able to offer long-term conservation benefits, such as increased prevalence or population trends for wide-ranging taxa [69]. Although multiple-use lands may be suboptimal in level of protection (and potentially habitat quality, but see below), their size may offer suitable area for wide-ranging species as well as dampen the effects of external pressures. These differences may equalize the effects of protected and multiple-use lands on species prevalence and population trend.

Third, highly mobile species may use a combination of protected, multiple-use, and undesignated lands during their annual cycle and thus may be exposed to a multitude of pressures (and similarly, multiple land categories) affecting population trend [70, 71]. Some species used in our analysis have large home ranges (see above) and/or are migratory for part of their annual cycle. Our study could not determine the extent to which species used protected or multiple-use lands, and thus, the associations between population trend and these areas may be conflated. Prevalence is likely affected by species mobility unless the species exhibits nomadism within or between breeding seasons (e.g., Henslow’s Sparrow, *Centronyx henslowii*; [72]).

Fourth, management strategies between protected lands and multiple-use lands, although different, may result in similar habitat quality on some lands. Not all lands under multiple-use management are used concurrently, to the same extent, or for the same purposes. In national forests, 9% (~73 million acres) are categorized as ‘reserve forests’ and are not managed for timber harvest, likewise, national forests also contain lands with ‘wilderness’ status [73]. Ecological communities in these and other multiple-use lands may progress to late seral stages (e.g., old-growth longleaf pine forests; [74]), similar to communities within protected lands. Furthermore, multiple-use lands may support some imperiled species populations as well as or better than protected lands because: (1) many multiple-use lands are large, thus offering suitable home range sizes and protection from external pressures (e.g., see above); (2) multiple-use lands are distributed broadly and may function to connect populations (e.g., national and state forests and recreation areas among the Appalachian mountains); (3) multiple-use lands contain a variety of ecological systems and may support a greater diversity of species than protected lands [11]; and (4) multiple-use lands experience more frequent anthropogenic disturbance events (that may be less common on protected lands) and thus offer a continuum of seral stages as well as more early successional habitat that supports high species diversity [75, 76]. Such factors (1) cannot be accounted for at the national scale; and (2) increase the capacity of multiple-use lands to protect biodiversity and potentially make it difficult to find differences between the effects of protected and multiple-use lands on population trends.

Fifth, some factors intrinsic to our dataset may have limited the identification of ecological responses. The imperiled species group, by its composition, may be hiding complexity that is obscuring patterns: (1) it may be difficult to detect differences in species trends when all trends are negative; and (2) many imperiled species are associated with grassland and aridland habitat, yet most of the eastern imperiled species are scrubland species, and thus, there may be complexity covered up by our grouping because the scrubland species would respond well to multiple-use management. Additionally, our approach minimized the likelihood of spurious relationships (see Methods), but those same efforts reduced the number of species included in the analyses. Although, these reductions may have buffered differences between protected and multiple-use lands, they should not have eliminated our ability to detect any difference between these groups. Another factor may be related to the road-based structure of the BBS route data collection. Some species elicit avoidance behaviors near roads, even within protected lands [77]; however, we feel that the percentage of avoidance would be small and its affect would be negligible and overcome by the number of species analyzed in this study. Furthermore, we must also consider that we do not have surveys that monitor population changes solely within protected areas. Lastly, we lack adequate characterization of the quality of habitat across both protected and multiple-use lands. However, even given these potential sources for noise in these data, we believe that the breadth and extent of this analysis was appropriate and sufficient to detect broad-scale, ecological signals related to species response to protected and multiple-use land management.

With the current population declines observed in many bird species, there is an increased importance in protected and multiple-use lands preventing extinction and maintaining avian diversity. We were able to show that both land categories do have a role in sustaining imperiled and non-imperiled bird species over the long term and across landscapes. Future research on the differences in habitat quality between protected and multiple-use lands would provide a better understanding of the role each plays in the maintaining bird populations across their annual cycle. Furthermore, the popularity of citizen science along with continued BBS surveys can provide invaluable data for future assessments of population trends and prevalence of birds within protected and multiple-use lands [78, 79].

## Conclusions

Conservation efforts continue to focus on protected lands as the primary tool to protect biodiversity [5, 7, 80]. Evaluating the effectiveness of this tool is critical so that supplemental or alternative actions can be implemented, if necessary [5, 10]. Our results suggest that the combination of protected and multiple-use lands is important for biodiversity. However, conservation cannot solely rely on protected lands to maintain avian biodiversity, perhaps in part because these lands are influenced by the external pressures of surrounding land-use, are too small and isolated and/or are inadequate for highly mobile species [8, 20, 21, 81].

Because undesignated lands make up 74% of the landscape in the contiguous US [17], these lands can have an important role in the conservation of biodiversity. However, a comprehensive and broad-scale characterization of these lands and analysis of their varied effects on species populations is required. To increase the efficacy of protected lands, a conservation management approach that strategically considers the roles and importance of all lands, including the tradeoffs associated with different land management strategies, will be necessary to fully protect and maintain biodiversity [82–84].

## Supporting information

S1 Fig Median prevalence based on the presence of protected, multiple-use, and undesignated lands. Median prevalence for imperiled species and non-imperiled species for Breeding Bird Survey (BBS) routes with ≥50% of protected, multiple-use, or undesignated land within a 2000-meter radius buffer surrounding routes. Data are presented by species group: Imperiled and Non-imperiled; by temporal subsets: long-term data (1966–2014; A, C, E) and short-term data (1993–2014; B, D, F); and by spatial subsets: CONUS (A, B), West (C, D), and East (E, F). West and East subsets were divided by the 98^th^. Brackets over bars show pairewise comparisons (e.g., the top bar shows the protected vs. undesignated comparison). Asterisks indicate significant differences between pairs based on Friedman’s chi-square test with post-hoc analysis. Significance was evaluated with p ≤ 0.10 for Friedman’s chi-square tests and p ≤ 0.03 with Bonferroni adjustment for post-hoc analysis. Error bars respresent ± SE. Specific results can be found in S4 Table.
(TIF)

S2 Fig Median population trend based on the presence of protected, multiple-use, and undesignated lands. Median prevalence for imperiled species and non-imperiled species for Breeding Bird Survey (BBS) routes with ≥50% of protected, multiple-use, or undesignated land within a 2000-meter radius buffer surrounding routes. Data are presented by species group: Imperiled and Non-imperiled; by temporal subsets: long-term data (1966–2014; A, C, E) and short-term data (1993–2014; B, D, F); and by spatial subsets: CONUS (A, B), West (C, D), and East (E, F). West and East subsets were divided by the 98^th^. Brackets over bars show pairewise comparisons (e.g., the top bar shows the protected vs. undesignated comparison). Asterisks indicate significant differences between pairs based on Friedman’s chi-square test with post-hoc analysis. Significance was evaluated with p ≤ 0.10 for Friedman’s chi-square tests and p ≤ 0.03 with Bonferroni adjustment for post-hoc analysis. Error bars respresent ± SE. Specific results can be found in S4 Table.
(TIF)

S3 Fig Comparison of linear models that included or excluded predictor variables for prevalence. ‘Predictor variables included’ indicates models which included proportional area of protected and multiple-use lands as predictor variables in the linear regression models to explain prevalence. ‘Competing models’ were those with delta AIC values <2, and models in which including or excluding the predictor variables did not change the delta AIC value. ‘Predictor variables excluded’ indicates models that did not include the proportional area variables. Additional covariates included proportion of developed areas, proportion of agricultural areas, median elevation of BBS route, total number of years BBS route was surveyed, and longitude and latitude at centroid of buffer. Data are presented by species group: Imperiled and Non-imperiled; by temporal subsets: long-term data (1966–2014; A, C, E) and short-term data (1993–2014; B, D, F); and by spatial subsets: CONUS (A, B), West (C, D), and East (E, F). West and East subsets were divided by the 98^th^.
(TIF)

S4 Fig Comparison of linear models that included or excluded predictor variables for population trends. ‘Predictor variables included’ indicates models which included proportional area of protected and multiple-use lands as predictor variables in the linear regression models to explain population trends. ‘Competing models’ were those with delta AIC values <2, and models in which including or excluding the predictor variables did not change the delta AIC value. ‘Predictor variables excluded’ indicates models that did not include the proportional area variables. Additional covariates included proportion of developed areas, proportion of agricultural areas, median elevation of BBS route, total number of years BBS route was surveyed, and longitude and latitude at centroid of buffer. Data are presented by species group: Imperiled and Non-imperiled; by temporal subsets: long-term data (1966–2014; A, C, E) and short-term data (1993–2014; B, D, F); and by spatial subsets: CONUS (A, B), West (C, D), and East (E, F).
(TIF)

S5 Fig Percent of species for which prevalence was positively associated with land category. Species prevalence may be positively associated with both land categories. Horizontal lines at 50% indicate the null model (i.e., the percentage we would expect if bird prevalence were not influenced by proportion of protected or multiple-use lands). Breeding Bird Survey (BBS) routes were buffered using a 2000-meter radius. Data are presented by species group: Imperiled and Non-imperiled; by temporal subsets: long-term data (1966–2014; A, C, E) and short-term data (1993–2014; B, D, F); and by spatial subsets: CONUS (A, B), West (C, D), and East (E, F). West and East subsets were divided by the 98^th^. Asterisks indicate significant differences between pairs based on McNemar’s chi-square. Significance was evaluated with p ≤ 0.10. Specific results can be found in S5 Table.
(TIF)

S6 Fig Percent of species for which population trend was positively associated with land category. Species population trends may be positively associated with both land categories. Horizontal lines at 50% indicate the null model (i.e., the percentage we would expect if bird population trends were not influenced by proportion of protected or multiple-use lands). Breeding Bird Survey (BBS) routes were buffered using a 2000-meter radius. Data are presented by species group: Imperiled and Non-imperiled; by temporal subsets: long-term data (1966–2014; A, C, E) and short-term data (1993–2014; B, D, F); and by spatial subsets: CONUS (A, B), West (C, D), and East (E, F). West and East subsets were divided by the 98^th^. Asterisks indicate significant differences between pairs based on McNemar’s chi-square. Significance was evaluated with p ≤ 0.10. Specific results can be found in S5 Table.
(TIF)

S7 Fig Species for which prevalence was more positively associated with proportion of protected land than with proportion multiple-use land. Bird Survey (BBS) routes were buffered using a 2000-meter radius. Data are presented by species group: Imperiled and Non-imperiled; by temporal subsets: long-term data (1966–2014; A, C, E) and short-term data (1993–2014; B, D, F); and by spatial subsets: CONUS (A, B), West (C, D), and East (E, F). West and East subsets were divided by the 98^th^. Significance was evaluated with *P* ≤ 0.10 using a two-tailed Chi-squared test. Specific results can be found in S6 Table.
(TIF)

S8 Fig Species for which population trend was more positively associated with proportion of protected land than proportion with multiple-use land. Bird Survey (BBS) routes were buffered using a 2000-meter radius. Data are presented by species group: Imperiled and Non-imperiled; by temporal subsets: long-term data (1966–2014; A, C, E) and short-term data (1993–2014; B, D, F); and by spatial subsets: CONUS (A, B), West (C, D), and East (E, F). West and East subsets were divided by the 98^th^. Significance was evaluated with *P* ≤ 0.10 using a two-tailed Chi-squared test. Specific results can be found in S6 Table.
(TIF)

S1 Table Percent of buffer area of Breeding Bird Survey routes in protected and multiple-use lands. The 2000-meter radius buffers were categorized as containing 100%, 75%, or 50% of protected and multiple-use land.
(DOCX)

S2 Table Imperiled species prevalence and population trend relationships with proportion of protected and multiple use lands. Proportional area was calculated within 2000-meter radius buffer surrounding Breeding Bird Survey (BBS) routes. A plus sign (+) indicates that prevalence or population trend for a bird species was positively associated with the proportional area of protected or multiple-use land within the buffer. A negative sign (–) indicates that prevalence or population trend for a bird species was negatively associated with the proportional area of protected or multiple-use land within the buffer. Duplicate names indicate multiple forms, as designated by the BBS. NA indicates that the species was not used in the analysis as a result of criteria restrictions. Species are listed in alphabetical order by common name.
(DOCX)

S3 Table Non-imperiled species prevalence and population trend relationships with proportion of protected and multiple use lands. Proportional area was calculated within 2000-meter radius buffer surrounding Breeding Bird Survey (BBS) routes. A plus sign (+) indicates that prevalence or population trend for a bird species was positively associated with the proportional area of protected or multiple-use land within the buffer. A negative sign (–) indicates that prevalence or population trend for a bird species was negatively associated with the proportional area of protected or multiple-use land within the buffer. Duplicate names indicate multiple forms, as designated by the BBS. NA indicates that the species was not used in the analysis as a result of criteria restrictions. Species are listed in alphabetical order by common name.
(DOCX)

S4 Table Median prevalence and population trends based on the presence of protected, multiple-use, and undesignated lands. Median prevalence and population trends for imperiled species and non-imperiled species for Breeding Bird Survey (BBS) routes with ≥50% of protected, multiple-use, or undesignated land within a 2000-meter radius buffer surrounding routes. Data are presented by species group, (Imperiled and Non-imperiled), by temporal subsets (long-term data (1966–2014) and short-term data (1993–2014)), by spatial subsets (CONUS, West, and East). Asterisks indicate significant differences between pairs based on Friedman’s chi-square test with post-hoc analysis. Significance was evaluated with p ≤ 0.10 for Friedman’s chi-square tests and p ≤ 0.03 with Bonferroni adjustment for post-hoc analysis. See corresponding graphs in S1 and S2 Figs.
(DOCX)

S5 Table McNemar’s chi-square results. Columns, such as +Protected, +Multiple-use, show the number of species that were positively associated with proportion of these lands. See corresponding graphs in S5 and S6 Figs.
(DOCX)

S6 Table Two-tailed chi-square results. Bird Survey (BBS) routes were buffered using a 2000-meter radius. Differences were evaluated with a null hypothesis that the underlying probability of success is 0.5. Data are presented by species group: Imperiled and Non-imperiled; by temporal subsets: long-term data (1966–2014) and short-term data (1993–2014); and by spatial subsets: CONUS, West, and East. West and East subsets were divided by the 98^th^. Significance was evaluated with *P* ≤ 0.10 using a two-tailed chi-squared test. See S8 Fig for a graphical representation of these data. df = degrees of freedom. See corresponding graphs in S7 and S8 Figs.
(DOCX)
