# Supplementary figures and images for: Assessing the efficacy of protected and multiple-use lands for bird conservation in the U.S
Source: PLoS One. 2020 Sep 30;15(9):e0239184. doi: 10.1371/journal.pone.0239184 (PMC7526929; doi:10.1371/journal.pone.0239184)

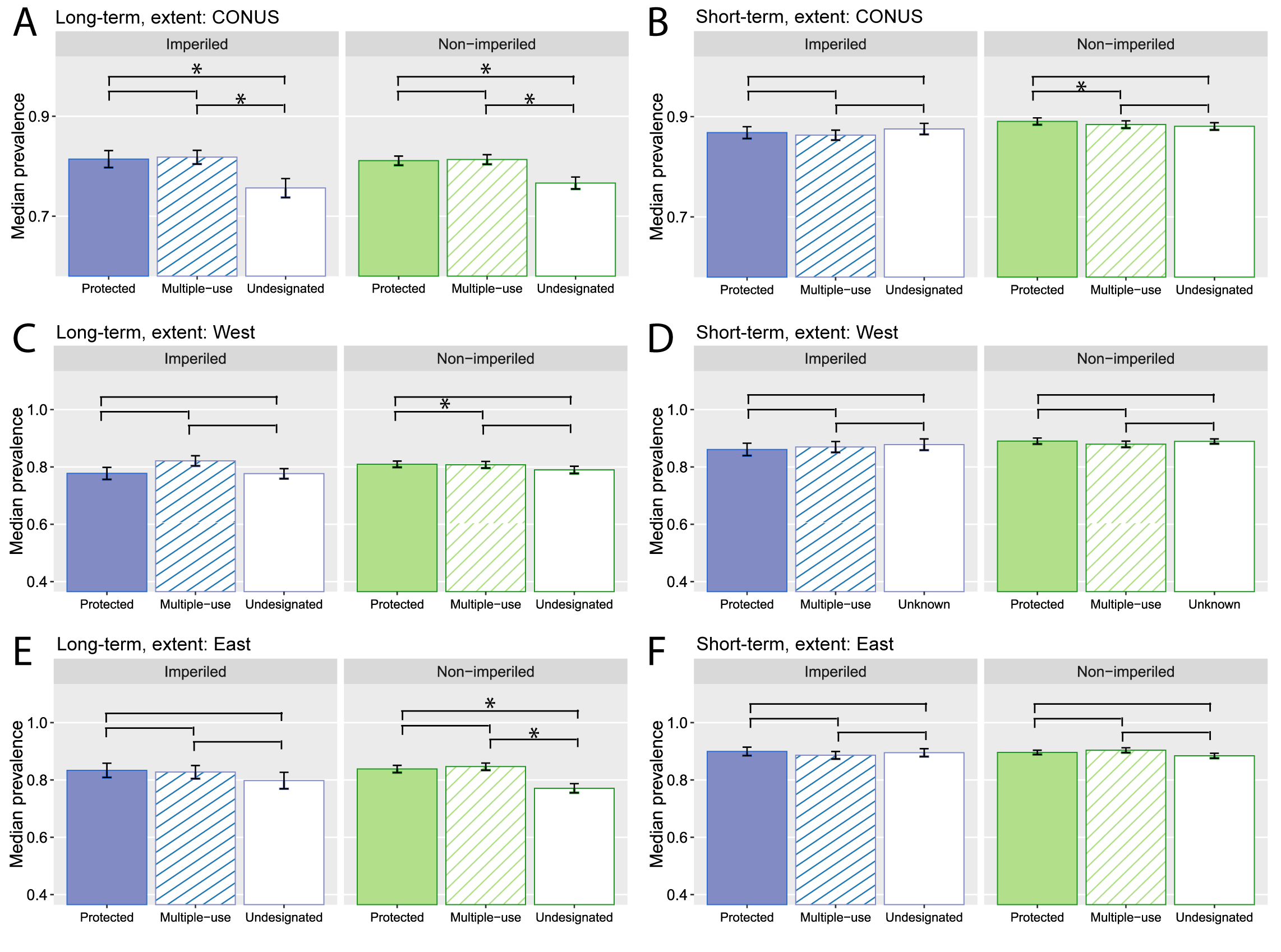

Supplement: S1 Fig — Median prevalence for imperiled species and non-imperiled species for Breeding Bird Survey (BBS) routes with ≥50% of protected, multiple-use, or undesignated land within a 2000-meter radius buffer surrounding routes. Data are presented by species group: Imperiled and Non-imperiled; by temporal subsets: long-term data (1966–2014; A, C, E) and short-term data (1993–2014; B, D, F); and by spatial subsets: CONUS (A, B), West (C, D), and East (E, F). West and East subsets were divided by the 98th. Brackets over bars show pairewise comparisons (e.g., the top bar shows the protected vs. undesignated comparison). Asterisks indicate significant differences between pairs based on Friedman’s chi-square test with post-hoc analysis. Significance was evaluated with p ≤ 0.10 for Friedman’s chi-square tests and p ≤ 0.03 with Bonferroni adjustment for post-hoc analysis. Error bars respresent ± SE. Specific results can be found in S4 Table. (TIF) [file pone.0239184.s001.tif]

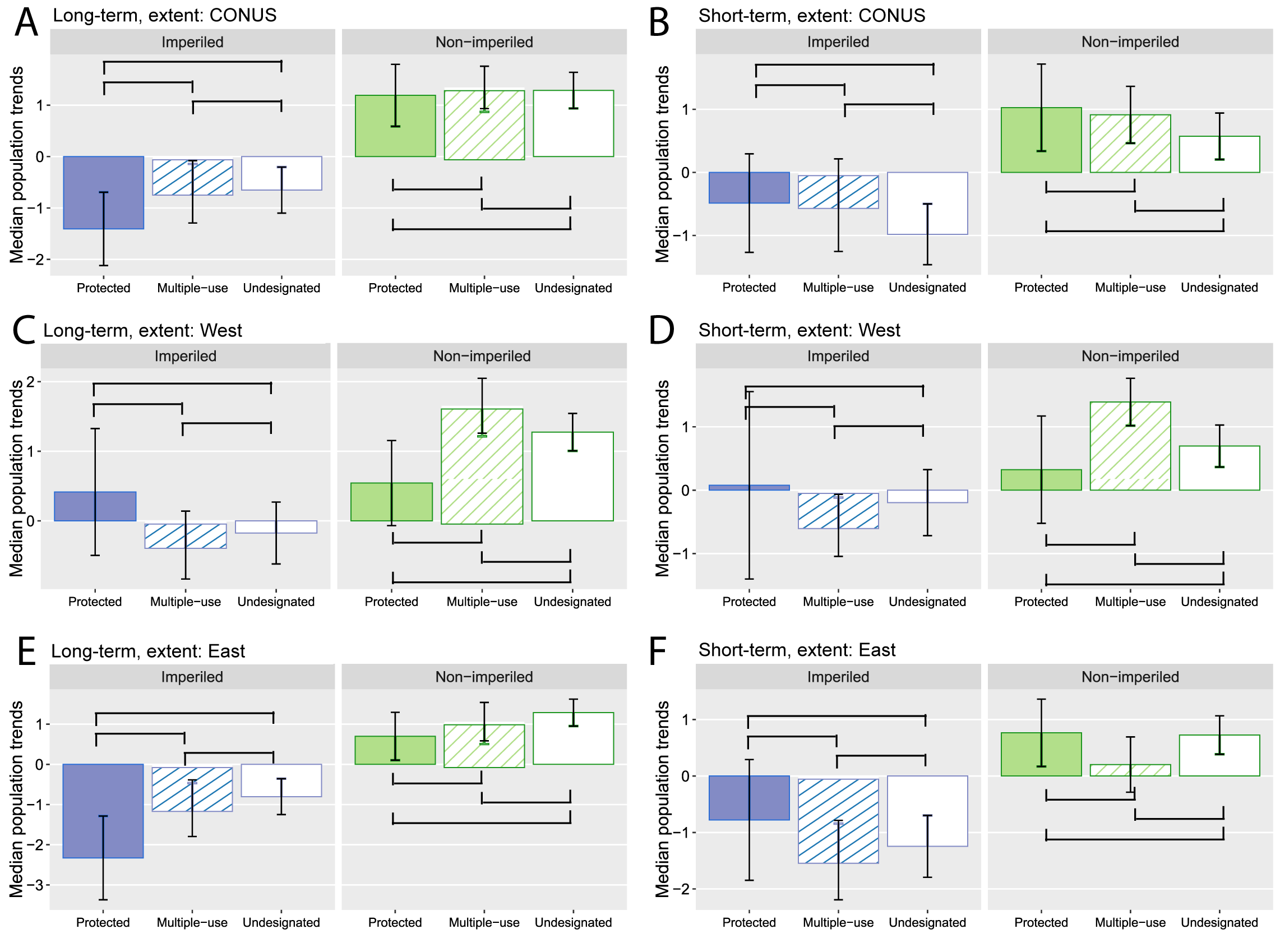

Supplement: S2 Fig — Median prevalence for imperiled species and non-imperiled species for Breeding Bird Survey (BBS) routes with ≥50% of protected, multiple-use, or undesignated land within a 2000-meter radius buffer surrounding routes. Data are presented by species group: Imperiled and Non-imperiled; by temporal subsets: long-term data (1966–2014; A, C, E) and short-term data (1993–2014; B, D, F); and by spatial subsets: CONUS (A, B), West (C, D), and East (E, F). West and East subsets were divided by the 98th. Brackets over bars show pairewise comparisons (e.g., the top bar shows the protected vs. undesignated comparison). Asterisks indicate significant differences between pairs based on Friedman’s chi-square test with post-hoc analysis. Significance was evaluated with p ≤ 0.10 for Friedman’s chi-square tests and p ≤ 0.03 with Bonferroni adjustment for post-hoc analysis. Error bars respresent ± SE. Specific results can be found in S4 Table. (TIF) [file pone.0239184.s002.tif]

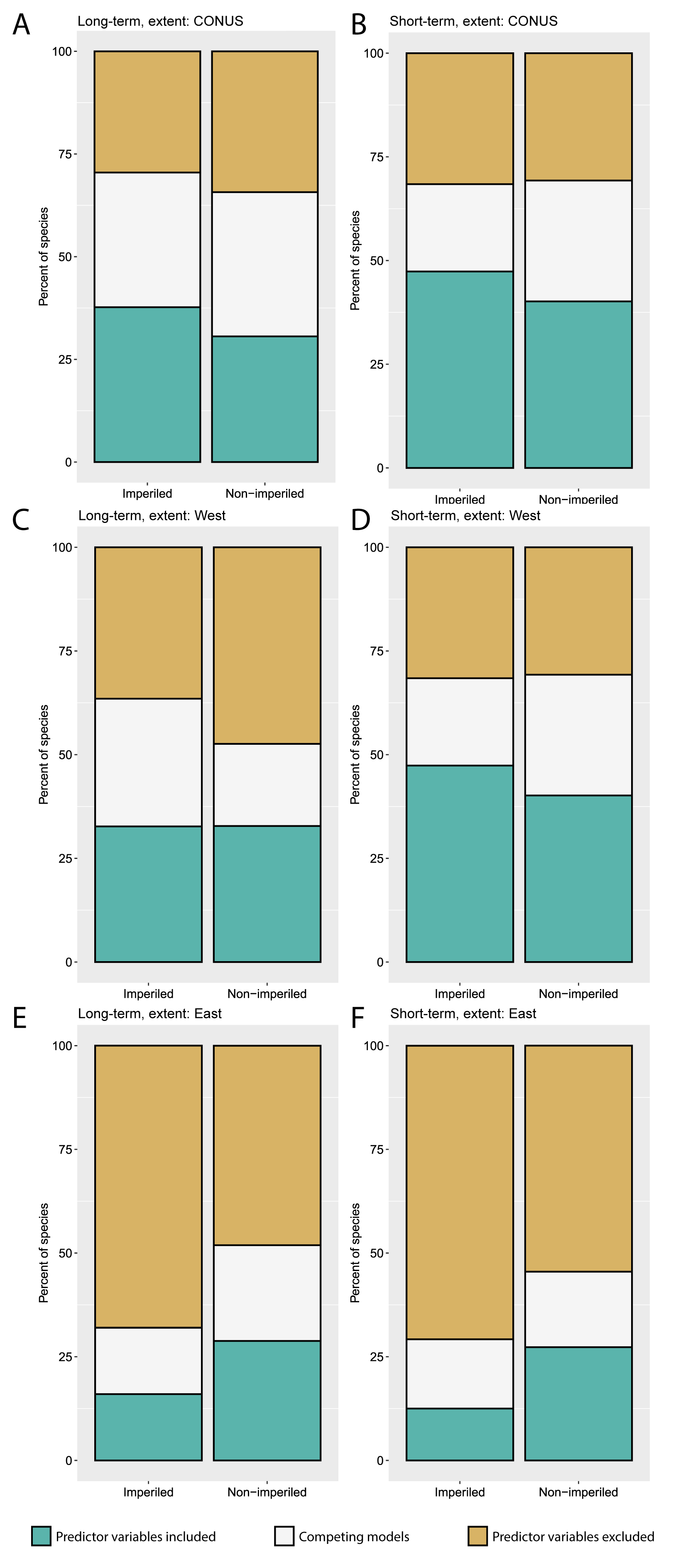

Supplement: S3 Fig — ‘Predictor variables included’ indicates models which included proportional area of protected and multiple-use lands as predictor variables in the linear regression models to explain prevalence. ‘Competing models’ were those with delta AIC values <2, and models in which including or excluding the predictor variables did not change the delta AIC value. ‘Predictor variables excluded’ indicates models that did not include the proportional area variables. Additional covariates included proportion of developed areas, proportion of agricultural areas, median elevation of BBS route, total number of years BBS route was surveyed, and longitude and latitude at centroid of buffer. Data are presented by species group: Imperiled and Non-imperiled; by temporal subsets: long-term data (1966–2014; A, C, E) and short-term data (1993–2014; B, D, F); and by spatial subsets: CONUS (A, B), West (C, D), and East (E, F). West and East subsets were divided by the 98th. (TIF) [file pone.0239184.s003.tif]

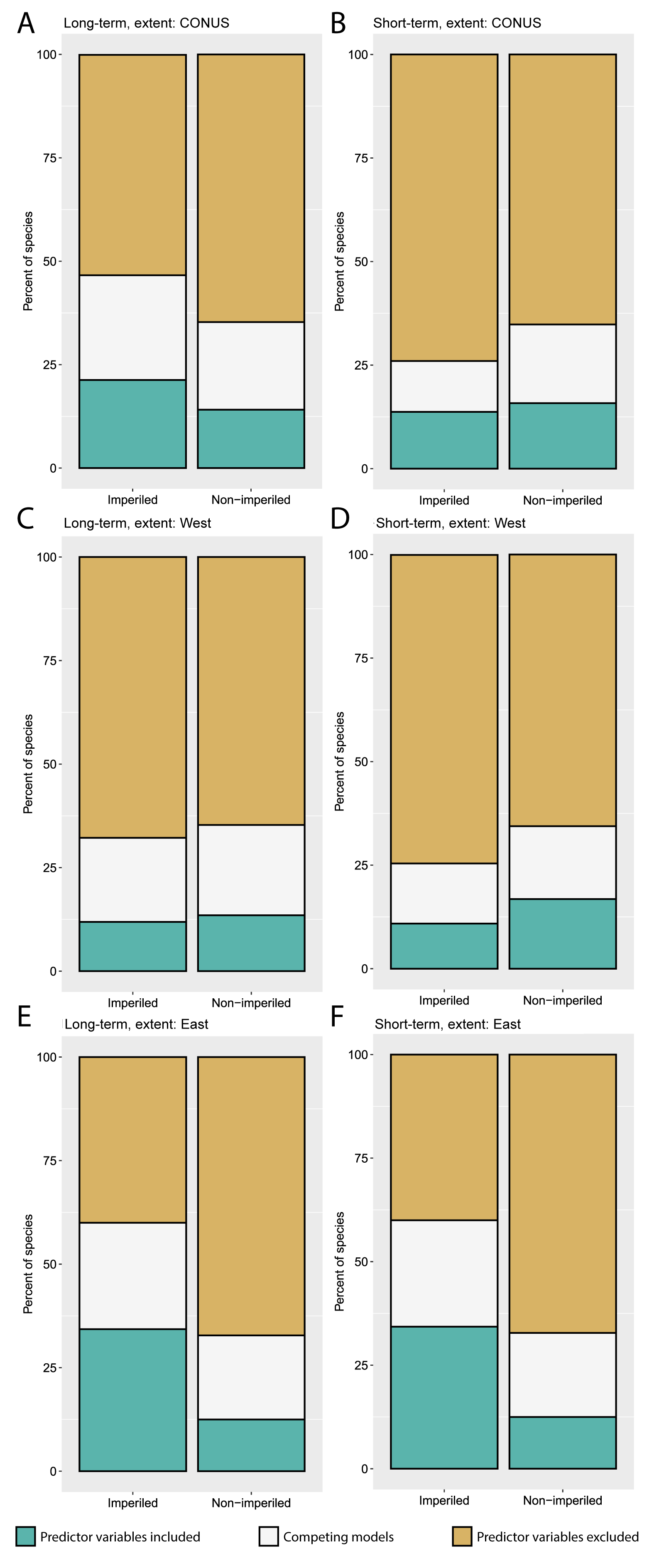

Supplement: S4 Fig — ‘Predictor variables included’ indicates models which included proportional area of protected and multiple-use lands as predictor variables in the linear regression models to explain population trends. ‘Competing models’ were those with delta AIC values <2, and models in which including or excluding the predictor variables did not change the delta AIC value. ‘Predictor variables excluded’ indicates models that did not include the proportional area variables. Additional covariates included proportion of developed areas, proportion of agricultural areas, median elevation of BBS route, total number of years BBS route was surveyed, and longitude and latitude at centroid of buffer. Data are presented by species group: Imperiled and Non-imperiled; by temporal subsets: long-term data (1966–2014; A, C, E) and short-term data (1993–2014; B, D, F); and by spatial subsets: CONUS (A, B), West (C, D), and East (E, F). (TIF) [file pone.0239184.s004.tif]

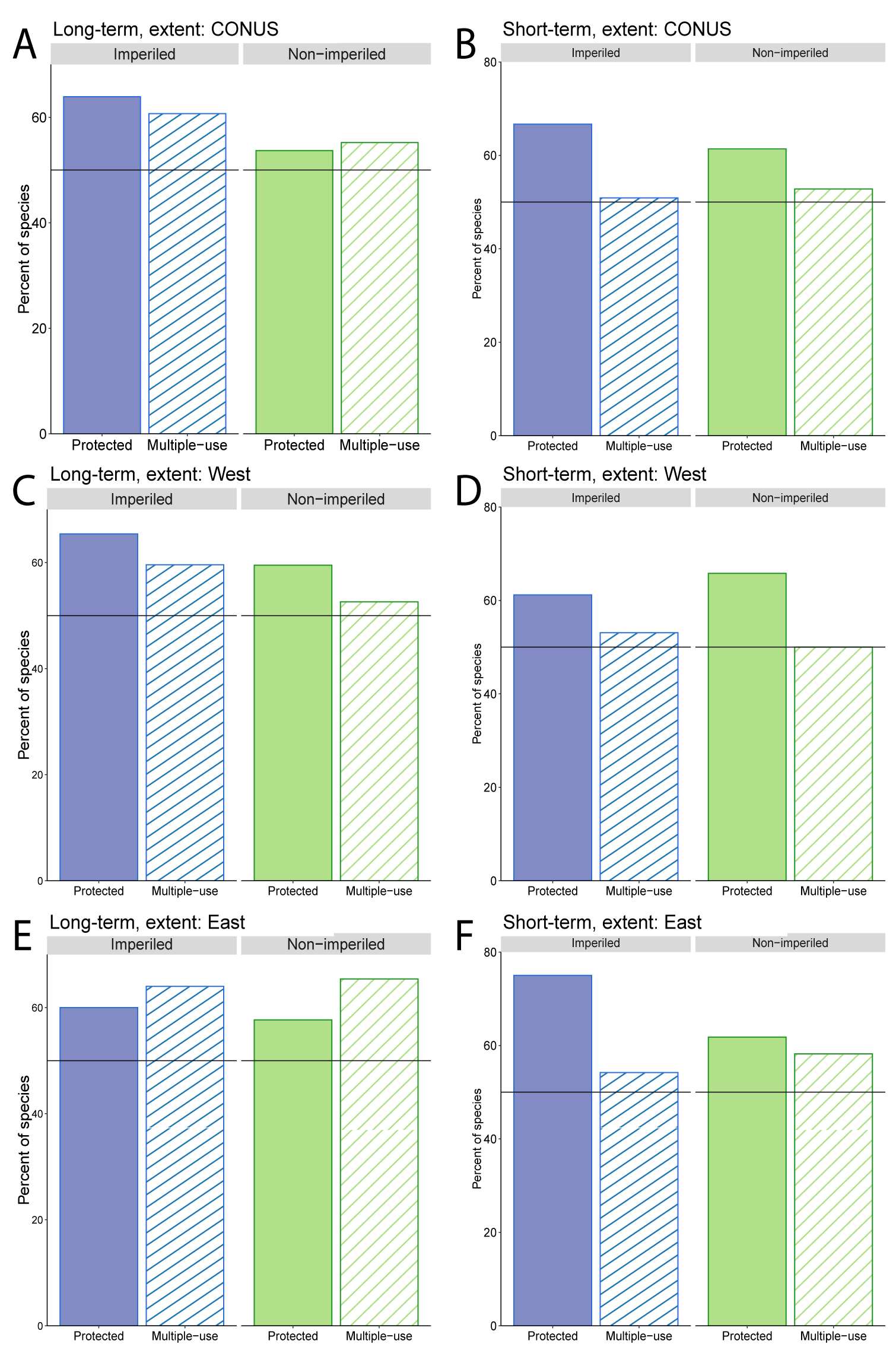

Supplement: S5 Fig — Species prevalence may be positively associated with both land categories. Horizontal lines at 50% indicate the null model (i.e., the percentage we would expect if bird prevalence were not influenced by proportion of protected or multiple-use lands). Breeding Bird Survey (BBS) routes were buffered using a 2000-meter radius. Data are presented by species group: Imperiled and Non-imperiled; by temporal subsets: long-term data (1966–2014; A, C, E) and short-term data (1993–2014; B, D, F); and by spatial subsets: CONUS (A, B), West (C, D), and East (E, F). West and East subsets were divided by the 98th. Asterisks indicate significant differences between pairs based on McNemar’s chi-square. Significance was evaluated with p ≤ 0.10. Specific results can be found in S5 Table. (TIF) [file pone.0239184.s005.tif]

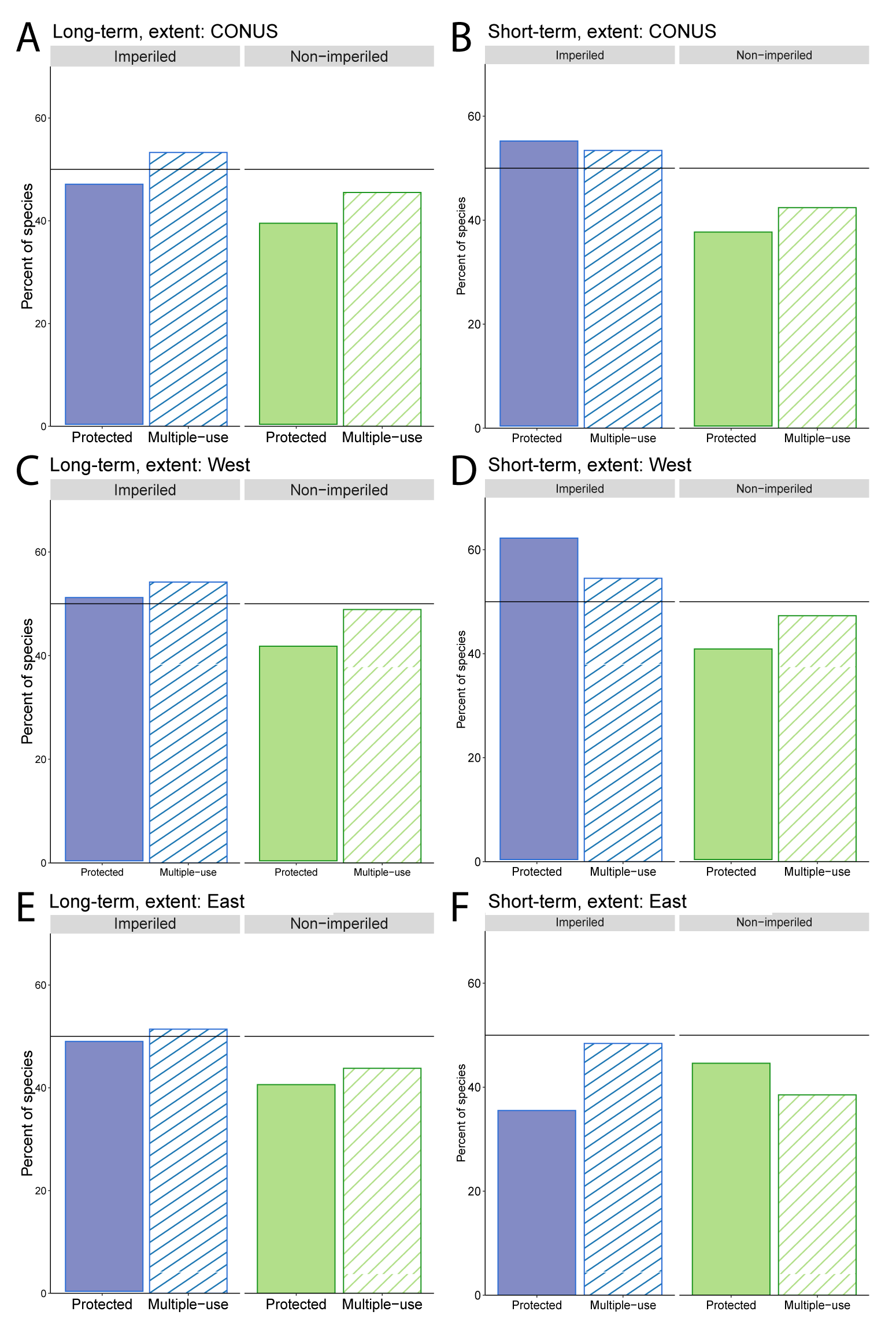

Supplement: S6 Fig — Species population trends may be positively associated with both land categories. Horizontal lines at 50% indicate the null model (i.e., the percentage we would expect if bird population trends were not influenced by proportion of protected or multiple-use lands). Breeding Bird Survey (BBS) routes were buffered using a 2000-meter radius. Data are presented by species group: Imperiled and Non-imperiled; by temporal subsets: long-term data (1966–2014; A, C, E) and short-term data (1993–2014; B, D, F); and by spatial subsets: CONUS (A, B), West (C, D), and East (E, F). West and East subsets were divided by the 98th. Asterisks indicate significant differences between pairs based on McNemar’s chi-square. Significance was evaluated with p ≤ 0.10. Specific results can be found in S5 Table. (TIF) [file pone.0239184.s006.tif]

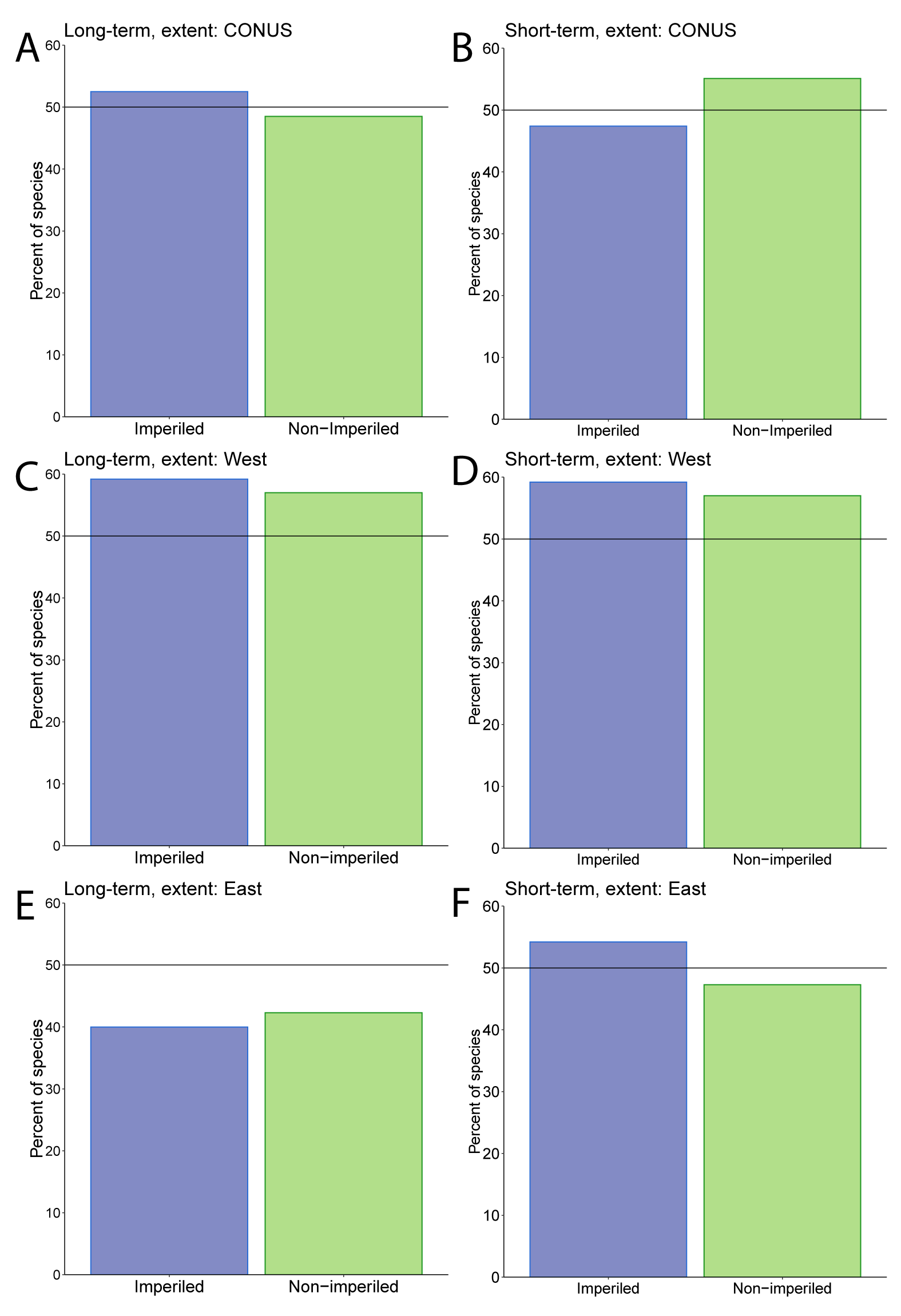

Supplement: S7 Fig — Bird Survey (BBS) routes were buffered using a 2000-meter radius. Data are presented by species group: Imperiled and Non-imperiled; by temporal subsets: long-term data (1966–2014; A, C, E) and short-term data (1993–2014; B, D, F); and by spatial subsets: CONUS (A, B), West (C, D), and East (E, F). West and East subsets were divided by the 98th. Significance was evaluated with P ≤ 0.10 using a two-tailed Chi-squared test. Specific results can be found in S6 Table. (TIF) [file pone.0239184.s007.tif]

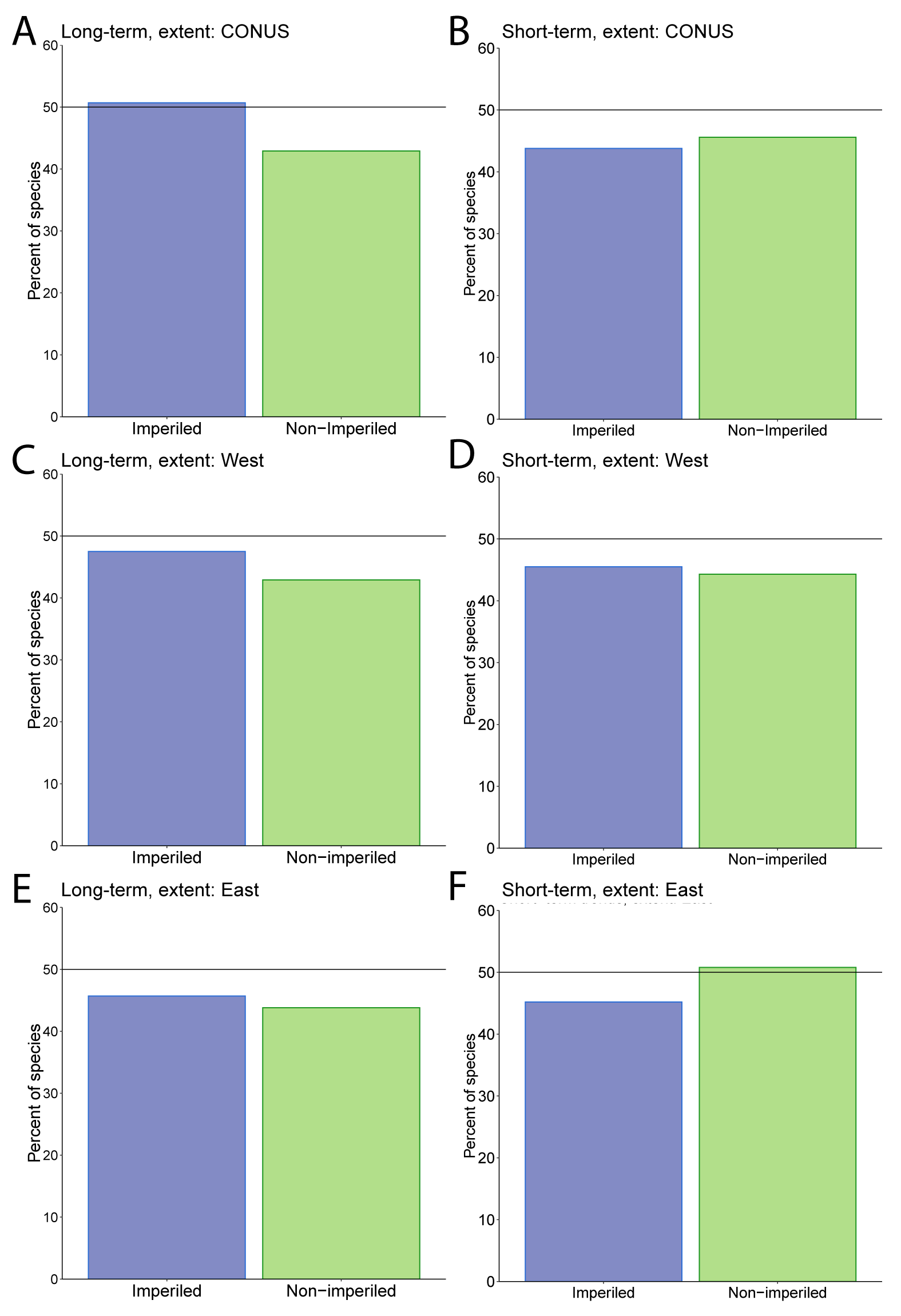

Supplement: S8 Fig — Bird Survey (BBS) routes were buffered using a 2000-meter radius. Data are presented by species group: Imperiled and Non-imperiled; by temporal subsets: long-term data (1966–2014; A, C, E) and short-term data (1993–2014; B, D, F); and by spatial subsets: CONUS (A, B), West (C, D), and East (E, F). West and East subsets were divided by the 98th. Significance was evaluated with P ≤ 0.10 using a two-tailed Chi-squared test. Specific results can be found in S6 Table. (TIF) [file pone.0239184.s008.tif]
