# Supplementary material for: Assessing the efficacy of protected and multiple-use lands for bird conservation in the U.S
Source: PLoS One. 2020 Sep 30;15(9):e0239184. doi: 10.1371/journal.pone.0239184 (PMC7526929; doi:10.1371/journal.pone.0239184)
Supplement: S1 Table — The 2000-meter radius buffers were categorized as containing 100%, 75%, or 50% of protected and multiple-use land. (DOCX) [file pone.0239184.s009.docx]

**S1 Table. Percent of buffer area of Breeding Bird Survey (BBS) routes used in analysis of species prevalence and population trends.** The 2000-meter radius buffers were categorized as containing 100%, 75%, or 50% of protected and multiple-use land.

| Percent of buffer area | Prevalence | | Population trend | |
| --- | --- | --- | --- | --- |
|  | Protected | Multiple-use | Protected | Multiple-use |
| 100 | 0.3 | 0.4 | 0.3 | 0.3 |
| 75 | 2.0 | 8.3 | 1.8 | 7.5 |
| 50 | 4.2 | 16.9 | 3.8 | 15.5 |
