# Supplementary material for: Assessing the efficacy of protected and multiple-use lands for bird conservation in the U.S
Source: PLoS One. 2020 Sep 30;15(9):e0239184. doi: 10.1371/journal.pone.0239184 (PMC7526929; doi:10.1371/journal.pone.0239184)
Supplement: S2 Table — Proportional area was calculated within 2000-meter radius buffer surrounding Breeding Bird Survey (BBS) routes. A plus sign (+) indicates that prevalence or population trend for a bird species was positively associated with the proportional area of protected or multiple-use land within the buffer. A negative sign (–) indicates that prevalence or population trend for a bird species was negatively associated with the proportional area of protected or multiple-use land within the buffer. Duplicate names indicate multiple forms, as designated by the BBS. NA indicates that the species was not used in the analysis as a result of criteria restrictions. Species are listed in alphabetical order by common name. (DOCX) [file pone.0239184.s010.docx]

**S2 Table. Imperiled species prevalence and population trend relationships with proportional area of protected and multiple use areas.** Proportional area was calculated within 2000-meter radius buffer surrounding Breeding Bird Survey (BBS) routes. A plus sign (+) indicates that prevalence or population trend for a bird species was positively associated with the proportional area of protected or multiple-use land within the buffer. A negative sign (–) indicates that prevalence or population trend for a bird species was negatively associated with the proportional area of protected or multiple-use land within the buffer. Duplicate names indicate multiple forms, as designated by the BBS. NA indicates that the species was not used in the analysis as a result of criteria restrictions. Species are listed in alphabetical order by common name.

| Species common name | Species scientific name | Prevalence^a^ | | Population trend^b^ | |
| --- | --- | --- | --- | --- | --- |
|  |  | Protected | Multiple-use | Protected | Multiple-use |
| Allen's hummingbird | *Selasphorus sasin* | + | - | - | - |
| American bittern | *Botaurus lentiginosus* | + | - | - | + |
| American oystercatcher | *Haematopus palliatus* | NA | NA | + | - |
| Bald eagle | *Haliaeetus leucocephalu* | + | - | - | + |
| Bay-breasted warbler | *Setophaga castanea* | - | + | - | + |
| Bendire's thrasher | *Toxostoma bendirei* | + | - | + | - |
| Black-chinned sparrow | *Spizella atrogularis* | + | + | - | + |
| Black-crowned night-heron | *Nycticorax nycticorax* | + | + | + | + |
| Black-throated gray warbler | *Setophaga nigrescens* | + | + | + | - |
| Black-throated green warbler | *Setophaga virens* | + | + | - | - |
| Black skimmer | *Rynchops niger* | - | + | - | - |
| Black swift | *Cypseloides niger* | NA | NA | + | + |
| Boreal chickadee | *Poecile hudsonicus* | NA | NA | - | + |
| Calliope hummingbird | *Stellula calliope* | - | + | - | + |
| Canada warbler | *Cardellina canadensis* | + | + | - | + |
| Caspian tern | *Hydroprogne caspia* | + | - | + | - |
| Cassin's finch | *Carpodacus cassinii* | + | + | + | + |
| Cerulean warbler | *Setophaga cerulea* | + | - | + | + |
| Common tern | *Sterna hirundo* | - | + | + | + |
| Connecticut warbler | *Oporornis agilis* | - | + | + | + |
| Costa's hummingbird | *Calypte costae* | + | + | + | + |
| Eared grebe | *Podiceps nigricollis* | - | - | + | + |
| Eastern whip-poor-will | *Caprimulgus vociferus* | + | + | + | - |
| Florida scrub-jay | *Aphelocoma coerulescens* | NA | NA | + | + |
| Gila woodpecker | *Melanerpes uropygialis* | + | + | - | + |
| Gilded flicker | *Colaptes chrysoides* | + | - | - | - |
| Grace's warbler | *Setophaga graciae* | - | + | + | - |
| Gray vireo | *Vireo vicinior* | + | - | - | + |
| Green-tailed towhee | *Pipilo chlorurus* | - | - | - | - |
| Gull-billed tern | *Gelochelidon nilotica* | + | - | + | + |
| Juniper titmouse | *Baeolophus ridgwayi* | + | + | - | - |
| Lawrence's goldfinch | *Spinus lawrencei* | - | - | + | - |
| Least bittern | *Ixobrychus exilis* | + | - | - | - |
| Least tern | *Sternula antillarum* | - | + | - | - |
| Limpkin | *Aramus guarauna* | NA | NA | - | - |
| Lucy's warbler | *Oreothlypis luciae* | - | - | + | + |
| Marsh wren | *Cistothorus palustris* | + | - | - | + |
| Northern goshawk | *Accipiter gentilis* | NA | NA | - | - |
| Nuttall's woodpecker | *Picoides nuttallii* | - | + | + | - |
| Oak titmouse | *Baeolophus inornatus* | - | + | - | + |
| Olive-sided flycatcher | *Contopus cooperi* | + | + | + | - |
| Olive warbler | *Peucedramus taeniatus* | NA | NA | + | + |
| Pelagic cormorant | *Phalacrocorax pelagicus* | NA | NA | - | - |
| Peregrine falcon | *Falco peregrinus* | NA | NA | + | + |
| Phainopepla | *Phainopepla nitens* | + | + | - | - |
| Pied-billed grebe | *Podilymbus podiceps* | + | - | - | + |
| Pine siskin | *Spinus pinus* | + | + | + | + |
| Pinyon jay | *Gymnorhinus cyanocephalu* | + | + | - | - |
| Prairie falcon | *Falco mexicanus* | - | + | + | + |
| Purple finch | *Carpodacus purpureus* | - | - | + | + |
| Red-cockaded woodpecker | *Picoides borealis* | + | + | + | + |
| Red-faced warbler | *Cardellina rubrifrons* | NA | NA | + | - |
| Red crossbill | *Loxia curvirostra* | + | + | - | + |
| Reddish egret | *Egretta rufescens* | NA | NA | + | + |
| Rock wren | *Salpinctes obsoletus* | + | + | - | - |
| Ruffed grouse | *Bonasa umbellus* | + | + | - | - |
| Rufous hummingbird | *Selasphorus rufus* | - | + | - | - |
| Rusty blackbird | *Euphagus carolinus* | NA | NA | - | + |
| Sage sparrow | *Amphispiza belli* | NA | NA | + | + |
| Seaside sparrow | *Ammodramus maritimus* | - | + | + | - |
| Snowy egret | *Egretta thula* | + | + | - | - |
| Snowy plover | *Charadrius nivosus* | NA | NA | + | - |
| Sooty grouse | *Dendragapus fuliginosus* | + | + | + | - |
| Spotted towhee | *Pipilo maculatus* | - | + | + | - |
| Swallow-tailed kite | *Elanoides forficatus* | - | - | + | + |
| Tricolored blackbird | *Agelaius tricolor* | + | - | - | + |
| Veery | *Catharus fuscescens* | - | - | - | + |
| Verdin | *Auriparus flaviceps* | + | + | - | + |
| Western grebe | *Aechmophorus occidentalis* | - | - | - | + |
| White-headed woodpecker | *Picoides albolarvatus* | + | + | - | - |
| Williamson's sapsucker | *Sphyrapicus thyroideus* | - | + | - | + |
| Wilson's warbler | *Cardellina pusilla* | + | - | + | - |
| Worm-eating warbler | *Helmitheros vermivorum* | + | + | - | - |
| Yellow-bellied sapsucker | *Sphyrapicus varius* | + | - | - | - |
| Yellow-billed magpie | *Pica nuttalli* | + | - | - | + |

^a^ *N* = 61

^b^*N*=75
