# Supplementary material for: Assessing the efficacy of protected and multiple-use lands for bird conservation in the U.S
Source: PLoS One. 2020 Sep 30;15(9):e0239184. doi: 10.1371/journal.pone.0239184 (PMC7526929; doi:10.1371/journal.pone.0239184)
Supplement: S3 Table — Proportional area was calculated within 2000-meter radius buffer surrounding Breeding Bird Survey (BBS) routes. A plus sign (+) indicates that prevalence or population trend for a bird species was positively associated with the proportional area of protected or multiple-use land within the buffer. A negative sign (–) indicates that prevalence or population trend for a bird species was negatively associated with the proportional area of protected or multiple-use land within the buffer. Duplicate names indicate multiple forms, as designated by the BBS. NA indicates that the species was not used in the analysis as a result of criteria restrictions. Species are listed in alphabetical order by common name. (DOCX) [file pone.0239184.s011.docx]

**S3 Table. Non-imperiled species prevalence and population trend relationships with proportional area of protected and multiple use areas.** Proportional area was calculated within 2000-meter radius buffer surrounding Breeding Bird Survey (BBS) routes. A plus sign (+) indicates that prevalence or population trend for a bird species was positively associated with the proportional area of protected or multiple-use land within the buffer. A negative sign (–) indicates that prevalence or population trend for a bird species was negatively associated with the proportional area of protected or multiple-use land within the buffer. Duplicate names indicate multiple forms, as designated by the BBS. NA indicates that the species was not used in the analysis as a result of criteria restrictions. Species are listed in alphabetical order by common name.

| Species common name | Species scientific name | Prevalence^a^ | | Population trend^b^ | |
| --- | --- | --- | --- | --- | --- |
|  |  | Protected | Multiple-use | Protected | Multiple-use |
| Abert's towhee | *Melozone aberti* | + | + | + | + |
| Accipiter hawk | *Accipiter sp.* | NA | NA | + | - |
| Acorn woodpecker | *Melanerpes formicivorus* | - | - | + | - |
| American coot | *Fulica americana* | + | + | + | + |
| American dipper | *Cinclus mexicanus* | - | - | + | + |
| American three-toed woodpecker | *Picoides dorsalis* | NA | NA | - | - |
| American white pelican | *Pelecanus erythrorhync* | + | + | - | + |
| Anhinga | *Anhinga anhinga* | + | + | - | + |
| Anna's hummingbird | *Calypte anna* | - | + | + | + |
| Ash-throated flycatcher | *Myiarchus cinerascens* | - | - | - | - |
| Band-tailed pigeon | *Patagioenas fasciata* | + | - | - | - |
| Barrow's goldeneye | *Bucephala islandica* | NA | NA | - | - |
| Black-and-white warbler | *Mniotilta varia* | - | + | - | - |
| Black-backed woodpecker | *Picoides arcticus* | NA | NA | + | + |
| Black-headed grosbeak | *Pheucticus melanocephal* | - | - | + | + |
| Black-necked stilt | *Himantopus mexicanus* | + | - | - | - |
| Black-tailed gnatcatcher | *Polioptila melanura* | + | + | - | - |
| Black-throated blue warbler | *Setophaga caerulescens* | + | + | - | - |
| Black-throated sparrow | *Amphispiza bilineata* | + | + | - | - |
| Black phoebe | *Sayornis nigricans* | - | - | - | - |
| Blackburnian warbler | *Setophaga fusca* | + | + | + | + |
| Blackpoll warbler | *Setophaga striata* | NA | NA | + | - |
| Blue-headed vireo | *Vireo solitarius* | + | + | + | - |
| Boat-tailed grackle | *Quiscalus major* | + | + | + | - |
| Brewer's blackbird | *Euphagus cyanocephalu* | - | - | - | + |
| Broad-tailed hummingbird | *Selasphorus platycercus* | - | + | + | + |
| Brown-crested flycatcher | *Myiarchus tyrannulus* | + | + | - | + |
| Brown creeper | *Certhia americana* | + | + | - | - |
| Brown pelican | *Pelecanus occidentalis* | - | + | - | - |
| Bufflehead | *Bucephala albeola* | NA | NA | + | + |
| Bushtit | *Psaltriparus minimus* | - | - | + | - |
| California gull | *Larus californicus* | + | - | - | - |
| California quail | *Callipepla californica* | - | - | - | - |
| California thrasher | *Toxostoma redivivum* | - | + | - | - |
| California towhee | *Melozone crissalis* | - | - | - | + |
| Canyon wren | *Catherpes mexicanus* | + | + | - | + |
| Cape May warbler | *Setophaga tigrina* | + | + | - | - |
| Cassin's vireo | *Vireo cassinii* | - | - | + | - |
| Chestnut-backed chickadee | *Poecile rufescens* | + | + | - | - |
| Chestnut-sided warbler | *Setophaga pensylvanica* | - | - | - | - |
| Chukar | *Alectoris chukar* | - | - | + | + |
| Cinnamon teal | *Anas cyanoptera* | + | - | + | + |
| Clapper rail | *Rallus longirostris* | - | + | - | - |
| Clark's grebe | *Aechmophorus clarkii* | NA | NA | - | + |
| Clark's nutcracker | *Nucifraga columbiana* | + | + | - | - |
| Common gallinule | *Gallinula galeata* | + | - | - | + |
| Common goldeneye | *Bucephala clangula* | NA | NA | - | + |
| Common loon | *Gavia immer* | + | - | + | - |
| Common merganser | *Mergus merganser* | + | - | + | + |
| Common poorwill | *Phalaenoptilus nuttallii* | + | + | - | - |
| Common Raven | *Corvus corax* | + | - | - | - |
| Cordilleran flycatcher | *Empidonax occidentalis* | + | - | + | - |
| Crissal thrasher | *Toxostoma crissale* | + | - | - | - |
| Dark-eyed junco | *Junco hyemalis* | NA | NA | + | + |
| Dark-eyed junco | *Junco hyemalis caniceps* | + | + | - | - |
| Dark-eyed junco | *Junco hyemalis hyemalis* | + | + | - | + |
| Dark-eyed junco | *Junco hyemalis oreganus* | - | + | - | + |
| Double-crested cormorant | *Phalacrocorax auritus* | + | + | - | - |
| Dusky flycatcher | *Empidonax oberholseri* | - | + | - | - |
| Empidonax flycatcher | *Empidonax sp.* | - | - | - | + |
| Evening grosbeak | *Coccothraustes vespertinus* | + | + | + | + |
| Forster's tern | *Sterna forsteri* | - | - | - | + |
| Fox sparrow | *Passerella iliaca* | + | + | + | + |
| Franklin's gull | *Leucophaeus pipixcan* | + | - | - | - |
| Gadwall | *Anas strepera* | + | + | + | + |
| Gambel's quail | *Callipepla gambelii* | - | - | + | + |
| Glaucous-winged gull | *Larus glaucescens* | - | - | - | - |
| Glossy ibis | *Plegadis falcinellus* | + | - | - | + |
| Golden-crowned kinglet | *Regulus satrapa* | + | + | + | + |
| Gray flycatcher | *Empidonax wrightii* | - | - | - | - |
| Gray jay | *Perisoreus canadensis* | + | + | - | - |
| Gray kingbird | *Tyrannus dominicensis* | NA | NA | + | + |
| Great black-backed gull | *Larus marinus* | + | + | - | - |
| Hammond's flycatcher | *Empidonax hammondii* | + | + | + | + |
| Hepatic tanager | *Piranga flava* | - | + | - | + |
| Hermit thrush | *Catharus guttatus* | + | + | - | - |
| Hermit warbler | *Setophaga occidentalis* | - | + | - | + |
| Herring gull | *Larus argentatus* | + | + | - | + |
| Hooded merganser | *Lophodytes cucullatus* | NA | NA | + | + |
| Hutton's vireo | *Vireo huttoni* | - | - | + | + |
| King rail | *Rallus elegans* | + | + | - | - |
| Laughing gull | *Leucophaeus atricilla* | - | + | - | - |
| Lazuli bunting | *Passerina amoena* | - | - | - | + |
| Le Conte's thrasher | *Toxostoma lecontei* | - | - | + | + |
| Lesser goldfinch | *Spinus psaltria* | - | - | + | + |
| Lesser nighthawk | *Chordeiles acutipennis* | - | - | - | + |
| Lesser scaup | *Aythya affinis* | + | - | - | - |
| Lincoln's sparrow | *Melospiza lincolnii* | + | + | + | + |
| MacGillivray's warbler | *Geothlypis tolmiei* | - | + | - | - |
| Magnolia warbler | *Setophaga magnolia* | + | + | + | + |
| Merlin | *Falco columbarius* | NA | NA | - | + |
| Mottled duck | *Anas fulvigula* | + | - | - | - |
| Mountain bluebird | *Sialia currucoides* | - | - | - | - |
| Mountain chickadee | *Poecile gambeli* | - | - | + | - |
| Mountain quail | *Oreortyx pictus* | - | - | + | + |
| Mourning warbler | *Geothlypis philadelphia* | - | - | + | - |
| Mute swan | *Cygnus olor* | - | + | - | - |
| Nashville warbler | *Oreothlypis ruficapilla* | - | + | + | + |
| Northern pygmy-owl | *Glaucidium gnoma* | NA | NA | - | - |
| Northern waterthrush | *Parkesia noveboracens* | - | + | + | - |
| Orange-crowned warbler | *Oreothlypis celata* | - | - | - | - |
| Osprey | *Pandion haliaetus* | + | + | + | - |
| Ovenbird | *Seiurus aurocapilla* | + | + | + | - |
| Pacific-slope flycatcher | *Empidonax difficilis* | - | + | - | - |
| Pacific wren | *Troglodytes pacificus* | + | + | + | + |
| Palm warbler | *Setophaga palmarum* | NA | NA | + | - |
| Philadelphia vireo | *Vireo philadelphic* | NA | NA | + | + |
| Pine grosbeak | *Pinicola enucleator* | + | + | - | + |
| Pine warbler | *Setophaga pinus* | + | + | - | - |
| Purple gallinule | *Porphyrio martinica* | NA | NA | + | - |
| Pygmy nuthatch | *Sitta pygmaea* | + | + | - | - |
| Red-breasted nuthatch | *Sitta canadensis* | + | + | - | - |
| Red-breasted sapsucker | *Sphyrapicus ruber* | - | + | - | - |
| Red-naped sapsucker | *Sphyrapicus nuchalis* | - | - | - | - |
| Red-necked grebe | *Podiceps grisegena* | NA | NA | - | - |
| Red-shafted flicker | *Colaptes auratus cafer* | - | - | + | - |
| Redhead | *Aythya americana* | + | - | + | + |
| Ring-necked duck | *Aythya collaris* | - | - | + | - |
| Royal tern | *Thalasseus maximus* | - | + | - | - |
| Ruby-crowned kinglet | *Regulus calendula* | - | + | - | - |
| Ruddy duck | *Oxyura jamaicensis* | + | - | - | - |
| Sandhill crane | *Grus canadensis* | + | - | - | + |
| Scott's oriole | *Icterus parisorum* | + | + | - | - |
| Sora | *Porzana carolina* | + | - | + | + |
| Steller's jay | *Cyanocitta stelleri* | + | - | + | + |
| Swainson's thrush | *Catharus ustulatus* | - | + | - | + |
| Townsend's solitaire | *Myadestes townsendi* | + | + | - | - |
| Townsend's warbler | *Setophaga townsendi* | + | + | + | + |
| Tricolored heron | *Egretta tricolor* | - | - | - | - |
| Trumpeter swan | *Cygnus buccinator* | NA | NA | + | + |
| Varied thrush | *Ixoreus naevius* | + | + | + | + |
| Vaux's swift | *Chaetura vauxi* | + | + | - | - |
| Violet-green swallow | *Tachycineta thalassina* | + | - | - | + |
| Virginia rail | *Rallus limicola* | - | - | - | - |
| Western bluebird | *Sialia mexicana* | + | - | + | - |
| Western gull | *Larus occidentalis* | - | + | - | + |
| Western screech-owl | *Megascops kennicottii* | NA | NA | + | + |
| Western scrub-jay | *Aphelocoma californica* | - | - | + | + |
| Western tanager | *Piranga ludoviciana* | + | + | - | + |
| Western wood-pewee | *Contopus sordidulus* | - | - | + | - |
| White-crowned sparrow | *Zonotrichia leucophrys* | - | - | + | + |
| White-faced ibis | *Plegadis chihi* | + | - | - | - |
| White-tailed kite | *Elanus leucurus* | - | + | - | - |
| White-throated sparrow | *Zonotrichia albicollis* | - | - | + | + |
| White-throated swift | *Aeronautes saxatalis* | + | + | - | - |
| White-winged crossbill | *Loxia leucoptera* | NA | NA | + | + |
| White ibis | *Eudocimus albus* | - | + | - | - |
| Willet | *Tringa semipalmata* | + | + | - | - |
| Wilson's plover | *Charadrius wilsonia* | NA | NA | - | + |
| Winter wren | *Troglodytes hiemalis* | + | + | - | + |
| Wood stork | *Mycteria americana* | - | + | - | - |
| Woodpecker | *Woodpecker sp.* | NA | NA | - | - |
| Wrentit | *Chamaea fasciata* | - | + | - | - |
| Yellow-bellied flycatcher | *Empidonax flaviventris* | - | - | - | + |
| Yellow-rumped warbler | *Setophaga coronata coronata* | + | - | - | + |
| Yellow-rumped warbler | *Setophaga coronata auduboni* | + | + | - | - |

^a^ *N*=134

^b^*N*=156
