# Supplementary material for: Assessing the efficacy of protected and multiple-use lands for bird conservation in the U.S
Source: PLoS One. 2020 Sep 30;15(9):e0239184. doi: 10.1371/journal.pone.0239184 (PMC7526929; doi:10.1371/journal.pone.0239184)
Supplement: S4 Table — Median prevalence and population trends for imperiled species and non-imperiled species for Breeding Bird Survey (BBS) routes with ≥50% of protected, multiple-use, or undesignated land within a 2000-meter radius buffer surrounding routes. Data are presented by species group, (Imperiled and Non-imperiled), by temporal subsets (long-term data (1966–2014) and short-term data (1993–2014)), by spatial subsets (CONUS, West, and East). Asterisks indicate significant differences between pairs based on Friedman’s chi-square test with post-hoc analysis. Significance was evaluated with p ≤ 0.10 for Friedman’s chi-square tests and p ≤ 0.03 with Bonferroni adjustment for post-hoc analysis. See corresponding graphs in S1 and S2 Figs. (DOCX) [file pone.0239184.s012.docx]

**S4 Table. Comparison of prevalence and population trends within buffers with ≥50% of protected, multiple-use, or undesignated area.** Breeding Bird Survey (BBS) routes were buffered using a 2000-meter radius. Data are presented by temporal subsets: long-term data (1966-2014) and short-term data (1993-2014) and by spatial subsets: CONUS, West, and East. Asterisks indicate significant differences between pairs based on Friedman’s chi-square test with post-hoc analysis. Significance was evaluated with p ≤ 0.10 for Friedman’s chi-square tests and p ≤ 0.03 with Bonferroni adjustment for post-hoc analysis.

| Metric | Temporal subset | Spatial extent | Species group | Post-hoc analysis | Chi-square test | df | *p*-value |
| --- | --- | --- | --- | --- | --- | --- | --- |
| Prevalence | Long-term | CONUS | Imperiled |  | 11.4 | 2 | 0.003* |
|  |  |  |  | Protected – Multiple-use |  |  | 0.975 |
|  |  |  |  | Protected – Undesignated |  |  | 0.014* |
|  |  |  |  | Multiple-use –Undesignated |  |  | 0.007* |
|  |  |  | Non-imperiled |  | 28.0 | 2 | < 0.001* |
|  |  |  |  | Protected – Multiple-use |  |  | 0.923 |
|  |  |  |  | Protected – Undesignated |  |  | < 0.001* |
|  |  |  |  | Multiple-use –Undesignated |  |  | < 0.001* |
|  |  | West | Imperiled |  | 2.5 | 2 | 0.291 |
|  |  |  | Non-imperiled |  | 5.9 | 2 | 0.052* |
|  |  |  |  | Protected – Multiple-use |  |  | 0.033* |
|  |  |  |  | Protected – Undesignated |  |  | 0.111 |
|  |  |  |  | Multiple-use –Undesignated |  |  | 0.791 |
|  |  | East | Imperiled |  | 3.9 | 2 | 0.146 |
|  |  |  | Non-imperiled |  | 21.5 | 2 | < 0.001* |
|  |  |  |  | Protected – Multiple-use |  |  | 0.751 |
|  |  |  |  | Protected – Undesignated |  |  | < 0.001* |
|  |  |  |  | Multiple-use –Undesignated |  |  | < 0.001* |
|  |  |  |  |  |  |  |  |
|  | Short-term | CONUS | Imperiled |  | 3.0 | 2 | 0.227 |
|  |  |  | Non-imperiled |  | 8.0 | 2 | 0.019* |
|  |  |  |  | Protected – Multiple-use |  |  | 0.029* |
|  |  |  |  | Protected – Undesignated |  |  | 0.051 |
|  |  |  |  | Multiple-use –Undesignated |  |  | 0.975 |
|  |  | West | Imperiled |  | 2.0 | 2 | 0.368 |
|  |  |  | Non-imperiled |  | 5.2 | 2 | 0.073* |
|  |  |  |  | Protected – Multiple-use |  |  | 0.060 |
|  |  |  |  | Protected – Undesignated |  |  | 1 |
|  |  |  |  | Multiple-use –Undesignated |  |  | 0.486 |
|  |  | East | Imperiled |  | 1.6 | 2 | 0.449 |
|  |  |  | Non-imperiled |  | 3.0 | 2 | 0.219 |
|  |  |  |  | Protected – Multiple-use |  |  | 0.060 |
|  |  |  |  | Protected – Undesignated |  |  | 1 |
|  |  |  |  | Multiple-use –Undesignated |  |  | 0.486 |
| Population Trend | Long-term | CONUS | Imperiled |  | 1.6 | 2 | 0.455 |
|  |  |  | Non-imperiled |  | 0.6 | 2 | 0.742 |
|  |  | West | Imperiled |  | 0.3 | 2 | 0.867 |
|  |  |  | Non-imperiled |  | 1.6 | 2 | 0.446 |
|  |  | East | Imperiled |  | 5.2 | 2 | 0.074 |
|  |  |  |  | Protected – Multiple-use |  |  | 0.5 |
|  |  |  |  | Protected – Undesignated |  |  | 0.041 |
|  |  |  |  | Multiple-use –Undesignated |  |  | 0.311 |
|  |  |  | Non-imperiled |  | 2.6 | 2 | 0.273 |
|  |  |  |  |  |  |  |  |
|  | Short-term | CONUS | Imperiled |  | 0.96 | 2 | 0.618 |
|  |  |  | Non-imperiled |  | 0.36 | 2 | 0.837 |
|  |  | West | Imperiled |  | 0.11 | 2 | 0.949 |
|  |  |  | Non-imperiled |  | 4.47 | 2 | 0.107 |
|  |  | East | Imperiled |  | 3.1 | 2 | 0.213 |
|  |  |  | Non-imperiled |  | 1.0 | 2 | 0.609 |
