# Supplementary material for: Assessing the efficacy of protected and multiple-use lands for bird conservation in the U.S
Source: PLoS One. 2020 Sep 30;15(9):e0239184. doi: 10.1371/journal.pone.0239184 (PMC7526929; doi:10.1371/journal.pone.0239184)
Supplement: S5 Table — Columns, such as +Protected, +Multiple-use, show the number of species that were positively associated with proportion of these lands. See corresponding graphs in S5 and S6 Figs. (DOCX) [file pone.0239184.s013.docx]

**S5 Table. McNemar’s chi-square analysis results.** Columns, such as +Protected, +Multiple-use, show the number of species that were positively associated with proportion of these lands. Data are presented by species group: Imperiled and Non-imperiled; by temporal subsets: long-term data (1966-2014) and short-term data (1993-2014); and by spatial subsets: CONUS, West, and East. West and East subsets were divided by the 98^th^. Significance was evaluated with *P* ≤ 0.10. Asterisks indicate significant results. See corresponding graphs in S5 and S6 Figs.

| Metric | Temporal subset | Spatial extent | Species Group | + Protected,  + Multiple Use | + Protected,  - Multiple-use | - Protected,  + Multiple-Use | - Protected,  - Multiple-Use | McNemar’s chi-square | df | p-value |
| --- | --- | --- | --- | --- | --- | --- | --- | --- | --- | --- |
| Prevalence | Long-Term | CONUS | Imperiled | 23 | 16 | 14 | 8 | 0.03 | 1 | 0.855 |
|  |  |  | Non-imperiled | 49 | 23 | 25 | 37 | 0.02 | 1 | 0.885 |
|  |  | West | Imperiled | 20 | 14 | 11 | 7 | 0.16 | 1 | 0.689 |
|  |  |  | Non-imperiled | 46 | 24 | 16 | 31 | 1.23 | 1 | 0.268 |
|  |  | East | Imperiled | 9 | 6 | 7 | 3 | 0 | 1 | 1.000 |
|  |  |  | Non-imperiled | 23 | 7 | 11 | 11 | 0.5 | 1 | 0.480 |
|  | Short-term | CONUS | Imperiled | 24 | 14 | 5 | 14 | 3.37 | 1 | 0.067* |
|  |  |  | Non-imperiled | 49 | 29 | 18 | 31 | 2.13 | 1 | 0.145 |
|  |  | West | Imperiled | 17 | 13 | 9 | 10 | 0.41 | 1 | 0.522 |
|  |  |  | Non-imperiled | 46 | 29 | 11 | 28 | 7.23 | 1 | 0.007* |
|  |  | East | Imperiled | 10 | 8 | 3 | 3 | 1.45 | 1 | 0.228 |
|  |  |  | Non-imperiled | 27 | 7 | 5 | 16 | 0.08 | 1 | 0.773 |
| Population trends | Long-Term | CONUS | Imperiled | 20 | 15 | 20 | 20 | 0.46 | 1 | 0.499 |
|  |  |  | Non-imperiled | 41 | 20 | 30 | 65 | 1.62 | 1 | 0.203 |
|  |  | West | Imperiled | 18 | 12 | 14 | 15 | 0.04 | 1 | 0.845 |
|  |  |  | Non-imperiled | 41 | 14 | 24 | 54 | 2.13 | 1 | 0.144 |
|  |  | East | Imperiled | 11 | 6 | 7 | 11 | 0 | 1 | 1 |
|  |  |  | Non-imperiled | 12 | 14 | 16 | 22 | 0.03 | 1 | 0.855 |
|  | Short-term | CONUS | Imperiled | 25 | 16 | 15 | 18 | 0 | 1 | 1 |
|  |  |  | Non-imperiled | 37 | 22 | 30 | 69 | 0.94 | 1 | 0.332 |
|  |  | West | Imperiled | 22 | 12 | 8 | 13 | 0.45 | 1 | 0.502 |
|  |  |  | Non-imperiled | 38 | 15 | 24 | 54 | 1.64 | 1 | 0.200 |
|  |  | East | Imperiled | 5 | 6 | 10 | 10 | 0.56 | 1 | 0.453 |
|  |  |  | Non-imperiled | 12 | 17 | 13 | 23 | 0.3 | 1 | 0.584 |
