# Supplementary material for: Assessing the efficacy of protected and multiple-use lands for bird conservation in the U.S
Source: PLoS One. 2020 Sep 30;15(9):e0239184. doi: 10.1371/journal.pone.0239184 (PMC7526929; doi:10.1371/journal.pone.0239184)
Supplement: S6 Table — Bird Survey (BBS) routes were buffered using a 2000-meter radius. Differences were evaluated with a null hypothesis that the underlying probability of success is 0.5. Data are presented by species group: Imperiled and Non-imperiled; by temporal subsets: long-term data (1966–2014) and short-term data (1993–2014); and by spatial subsets: CONUS, West, and East. West and East subsets were divided by the 98th. Significance was evaluated with P ≤ 0.10 using a two-tailed chi-squared test. See S8 Fig for a graphical representation of these data. df = degrees of freedom. See corresponding graphs in S7 and S8 Figs. (DOCX) [file pone.0239184.s014.docx]

**S6 Table. Chi-square and *P*-value for percent of species for which population trends were more positively associated with proportion of protected land than with multiple-use land.** Bird Survey (BBS) routes were buffered using a 2000-meter radius. Differences were evaluated with a null hypothesis that the underlying probability of success is 0.5. Data are presented by species group: Imperiled and Non-imperiled; by temporal subsets: long-term data (1966-2014) and short-term data (1993-2014); and by spatial subsets: CONUS, West, and East. West and East subsets were divided by the 98^th^. Significance was evaluated with *P* ≤ 0.10 using a two-tailed chi-squared test. See S8 Fig for a graphical representation of these data. df = degrees of freedom. See corresponding graphs in S7 and S8 Figs.

| Metric | Temporal subset | Spatial extent | Species group | Chi-square test | df | *P*-value |
| --- | --- | --- | --- | --- | --- | --- |
| Prevalence | Long-term | CONUS | Imperiled | 0.07 | 1 | 0.798 |
|  |  |  | Non-imperiled | 0.07 | 1 | 0.796 |
|  |  | West | Imperiled | 0.48 | 1 | 0.488 |
|  |  |  | Non-imperiled | 0.01 | 1 | 0.926 |
|  |  | East | Imperiled | 0.64 | 1 | 0.424 |
|  |  |  | Non-imperiled | 0.94 | 1 | 0.332 |
|  | Short-term | CONUS | Imperiled | 0.07 | 1 | 0.791 |
|  |  |  | Non-imperiled | 1.13 | 1 | 0.287 |
|  |  | West | Imperiled | 1.31 | 1 | 0.253 |
|  |  |  | Non-imperiled | 1.97 | 1 | 0.160 |
|  |  | East | Imperiled | 0.04 | 1 | 0.838 |
|  |  |  | Non-imperiled | 0.07 | 1 | 0.787 |
| Population trend | Long-term | CONUS | Imperiled | 0 | 1 | 1 |
|  |  |  | Non-imperiled | 2.83 | 1 | 0.093* |
|  |  | West | Imperiled | 0.07 | 1 | 0.795 |
|  |  |  | Non-imperiled | 2.43 | 1 | 0.117 |
|  |  | East | Imperiled | 0.11 | 1 | 0.735 |
|  |  |  | Non-imperiled | 0.77 | 1 | 0.382 |
|  | Short-term | CONUS | Imperiled | 0.88 | 1 | 0.349 |
|  |  |  | Non-imperiled | 1.07 | 1 | 0.301 |
|  |  | West | Imperiled | 0.29 | 1 | 0.590 |
|  |  |  | Non-imperiled | 1.50 | 1 | 0.221 |
|  |  | East | Imperiled | 0.13 | 1 | 0.719 |
|  |  |  | Non-imperiled | 0 | 1 | 1 |
